# Exploring the anti‐cancer potential of dietary phytochemicals for the patients with breast cancer: A comprehensive review

**DOI:** 10.1002/cam4.5984

**Published:** 2023-05-03

**Authors:** Md Sohel, Suraiya Aktar, Partha Biswas, Md. Al Amin, Md. Arju Hossain, Nasim Ahmed, Md. Imrul Hasan Mim, Farhadul Islam, Abdullah Al Mamun

**Affiliations:** ^1^ Department of Biochemistry and Molecular Biology Primeasia University Dhaka Bangladesh; ^2^ Department of Biochemistry and Molecular Biology, Faculty of Life Science Mawlana Bhashani Science and Technology University Tangail Bangladesh; ^3^ Department of Biochemistry and Molecular Biology Rajshahi University Rajshahi Bangladesh; ^4^ Department of Genetic Engineering and Biotechnology, Faculty of Biological Science and Technology Jashore University of Science and Technology (JUST) Jashore Bangladesh; ^5^ Department of Biotechnology and Genetic Engineering, Faculty of Life Science Mawlana Bhashani Science and Technology University Tangail Bangladesh; ^6^ Department of Pharmacy Mawlana Bhashani Science and Technology University Tangail Bangladesh

**Keywords:** anti‐cancer mechanism, breast cancer, cancer treatment, natural products, phytochemicals, resistance

## Abstract

**Background:**

The most common and deadly cancer in female is breast cancer (BC) and new incidence and deaths related to this cancer are rising.

**Aims:**

Several issues, that is, high cost, toxicity, allergic reactions, less efficacy, multidrug resistance, and the economic cost of conventional anti‐cancer therapies, has prompted scientists to discover innovative approaches and new chemo‐preventive agents.

**Materials:**

Numerous studies are being conducted on plant‐based and dietary phytochemicals to discover new‐fangled and more advanced therapeutic approaches for BC management.

**Result:**

We have identified that natural compounds modulated many molecular mechanisms and cellular phenomena, including apoptosis, cell cycle progression, cell proliferation, angiogenesis and metastasis, up‐regulation of tumor‐suppressive genes, and down‐regulation of oncogenes, modulation of hypoxia, mammosphere formation, onco‐inflammation, enzymatic regulation, and epigenetic modifications in BC. We found that a number of signaling networks and their components such as PI3K/Akt/mTOR, MMP‐2 and 9, Wnt/‐catenin, PARP, MAPK, NF‐κB, Caspase‐3/8/9, Bax, Bcl2, Smad4, Notch1, STAT3, Nrf2, and ROS signaling can be regulated in cancer cells by phytochemicals. They induce up‐regulation of tumor inhibitor microRNAs, which have been highlighted as a key player for ani‐BC treatments followed by phytochemical supplementation.

**Conclusion:**

Therefore, this collection offers a sound foundation for further investigation into phytochemicals as a potential route for the development of anti‐cancer drugs in treating patients with BC.

## Graphical abstract

This review mainly focused the potential role of phytochemicals against breast cancer (BC) treatment with the molecular mechanism and cellular phenomenon. Altogether, this review also suggests that phytochemicals might be the good source to discover potential alternative and complementary chemotherapeutic agents for the treatment of BC due to their therapeutic advantages.

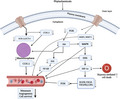

## 1 INTRODUCTION

Breast cancer (BC) is the most common and frequent malignancy among females, and it is the second most frequent carcinoma and a significant cause of cancer‐associated death worldwide. This cancer is a multifactorial disease and various factors, including demographic, oxidative stress, bacterial infection, reproductive, hormonal, hereditary, and lifestyle contribute to its occurrence.^1^ A number of conventional therapeutic options such as surgical resection, radiotherapy, chemo‐radiotherapies (e.g., adjuvant chemotherapies and neoadjuvant therapy), hormonal therapies, monoclonal antibodies, immunotherapy, and small molecular inhibitors are available for the patients with BC.^2^, ^3^ However, these therapeutic modalities have drawbacks, bearing side effects and toxicities. Thus, new approaches and strategies are needed to manage patients with BC effectively to minimize the limitations, such as increasing resistance to conventional therapeutics, side effects, and toxicities of existing treatment modalities. Interestingly, alternative medicines (with fewer side effects) for patients with BC, especially metastatic cancer, have been developed.

Phytochemicals are an essential natural resource for anti‐cancer medicine. They are safe, non‐toxic, cost‐effective, and readily available sources from villages to cities and underdeveloped to developed countries.^4^ Currently, medicinal plants or their derivatives account for about 70% of the anti‐cancer compounds, thus, playing the lead role in developing anti‐cancer drugs.^5^, ^6^ Initially, natural plant extracts have showed higher anti‐tumor responses and better pharmacological or bioactivity with less toxicity in patients with advanced BC (Table 1).^37^, ^38^, ^39^, ^40^ For example, anti‐cancer compounds from *Curcuma longa*, *Piper longum*, *Nigella sativa*, *Murrayakoenigii*, *Amora rohituka*, *Withania somnifera*, and *Dimocarpus longan* possess anti‐cancer activity against various cancers, especially anti‐BC properties.^8^, ^25^, ^36^, ^41^, ^42^, ^43^, ^44^ Latter specific phytochemicals have been identified as a new source of anti‐cancer agents from plant extract to decrease the negative effects of cancer chemotherapies in recent research.^45^, ^46^, ^47^, ^48^ These natural agents can target several BC‐related pathways and provide protective activity against breast malignancies, which play a significant role in preventing and managing patients with BC.^46^, ^49^ Several individual studies exhibited phytochemicals had anti‐cancer property through several mechanisms.^50^, ^51^, ^52^ However, a comprehensive summary on precise anti‐cancer mechanisms including apoptosis induction, cell cycle, and cell proliferation regulation, inhibition of angiogenesis and metastasis, regulating hypoxia‐inducible factor, suppressed mammosphere formation, onco‐inflammation inhibition, controlling enzyme activity, signal transduction regulation, epigenetic and immune regulation have not been reported collectively. Therefore, in this review, we have discussed various phytochemicals with their major sources, structure, and their possible anti‐cancer pathways in the BC, thereby providing an aggregative source of information on potential natural anti‐cancer resources.

**TABLE 1:** Summary of plants extract and their anti‐cancer activity in human breast cancer cell line.

Source/plant	Parts used	Working protocol			Anti‐cancer mechanism	Efficacy/dose	Ref.
		Methods	Extract used	Cell line			
*Ailanthus altissima*	Bark	Flow cytometry, RT‐PCR, western blot	Petroleum, dichloromethane	MCF‐7	↓ Cell proliferation ↑ Cell cycle arrest, apoptosis	0.5–8.0 μg/mL	^7^
*Amoora rohituka*	Leaf	FTIR analysis, phytochemical screening methods	Petroleum ether, ethyl acetate, methanol	MCF‐7	↓ Cell migration ↑ Apoptosis ↑ Cytotoxic effect	9.81 mg/mL	^8^
*Ardisia crispa*	Leaves	MTT assay, DPPH, ABTS assay	Ethyl acetate, aqueous	MCF‐7, MDA‐MB‐231	↓ Glucose uptake	57–100 μg/mL	^9^
*Baeckea frutescens*	Leaves extracts	Cytotoxity, glucose consumption assay	Ethanol, aqueous	MCF‐7 MDA‐MB‐231, MCF10A	↓ Cell viability, cell motility ↑ Cell cycle arrest, apoptosis	53 μg/mL	^10^
*Bryonia dioica*	Roots	Extracted, flow cytometry, staining, western blot	Aqueous	BL‐41	↑ Cell cycle arrest, apoptosis	15–63 g/mL	^11^
*Bulbine frutescens*	Bulb	Membrane potential, ROS, Notch promoter, western blot	Methanol, hexane	MDA‐MB‐231, T47D	↑ Cell cycle arrest, DNA repair, scavenge free radical	4.8–28.4 μg/mL	^12^
*Butea monosperma*	Bark fractions	MTT, clonogenic, neutral comet assay, flow cytometry	Methanol, hexane, chloroform, ethyl acetate	MCF‐7	↑ Inhibit proliferation, cell cycle arresting effect	44–213 mg/mL	^13^
*Cimicifuga dahurica*	Rizhomes	Extraction NMR, BrdU	70% ethanol	MCF‐7	↓ Oncogene expression cell proliferation ↑ Apoptosis induction	30 μM	^14^
*Decatropis bicolor*	Leaves	MTT assay, cell morphology analysis, western blot	Water, ethanol, acetone, hexane	MDA‐MB‐231	↑ Apoptosis induction	53.81 μg/mL	^15^
*Fagonia indica*	flower	Cytotoxicity, PARP, DNA fragmentation assay	EtOH	MCF‐7, MDA‐MB‐468	↑ Apoptosis	50–100 μM	^16^
*Garcinia oblongifoli*	Fruits, leaves	Cell viability, antioxidant	Methanol	MCF‐7	↑ Cytotoxic effect	1000 μg/mL	^17^
*Glycyrrhiza glabra*	Root	qRT‐PCR, western blots, DNA methylation analysis, immunostaining	Glabridin	Multiple cell line	↑ Anti‐tumor activity	0 or 20 mg/kg	^18^
*Hedyotis diffusa*	Leaves and shoots	Mitochondrial membrane potential, western blot	Methylanthraquinone	MCF‐7	↓ Cell growth ↑ Apoptosis	18.62 μM	^19^
*Lawsonia nermis*	Leaves	Chromatography, dynamic light scattering, UV–Vis spectroscopy	Alcoholic solution	MCF‐7	↑ Apoptosis, autophagy	1.5 μM	^20^
*Lotus corniculatus*	Leaves	MTT, PCR, wound healing assay	Ethyl acetate, methanol, water	MDA‐MB‐231, MCF‐7	↓ Cell migration, cancer‐related enzymatic activity	21.13 mg RE/g	^21^
*Lycium barbarum*	Fruit	Signaling mechanism test	NA	MCF‐7 cells	↓ Cancer‐related signaling, hypoxia condition	0.50 mg/mL	^22^
*Malus domestica*	Fruit	Western blot, cell cycle analysis	Acetone	MCF‐7, MDA‐MB‐231	↓ Enzyme activity, cell growth	10–80 mg/mL	^23^
*Morus alba*	Leaves	Anti‐proliferative radical scavenging assay	Methanol	MCF‐7	↑ Morphology change ↓ Cell proliferation	350 μg/mL	^24^
*Nigella sativa*	Seed	UV–visible spectroscopy, FT‐IR, SEM, EDX	Aqueous	MCF‐7	↑ Apoptosis ↓ Migration, adhesion, metastasis	1–200 μg/mL	^25^
*Platycodon grandiflorus*	Root	Cytotoxicity, flow cytometry, western	Platycodin D	MCF‐7	↑ Apoptosis	8 μg/mL	^26^
*Premna odorata*	Leaves	NMR, extraction, molecular modeling, proliferation, migration assay	70% ethanol	MCF‐7, BT‐ 474	↑ Cytotoxicity activity	13.3 μM	^27^
*Rabdosiae rubescens*	Whole part	Western blot analysis, immunohistochemistry analysis	Ethanol, water extract	MDA‐MB‐231 In vivo	↓ Growth migration, apoptosis	12 μg/mL	^28^
*Salpichroascandens*	Aerial parts	Extraction, cytotoxicity assay	Dichloromethane	MCF‐7, T47D	↓ Growth, cytotoxic activity	29–646 μM	^29^
*Salvia sclarea*	Plant	In vivo mice	n‐hexane/ethylacetate/methanol (1:1:1)	MCF‐7, T47D, ZR‐75‐1	↑ Anti‐proliferative, cytotoxicity activity	7.85 μg/day	^30^
*Salvia species*	N/A	Sulforhodamine B assay, chromatography	Ethanol	T47D, ZR‐75‐1, BT 474	↓ Aromatase enzyme	30 μg/mL	^31^
*Schisandra chinensis*	Seeds, leaves, and stems	Western blotting, Immunohistochemistry	Schisandrin A	MDA‐MB‐ 231, BT‐549	↑ Apoptosis induction, cell cycle arrest, cell cytotoxicity	134.21 ± 6.85 μM	^32^
*Scrophularia variegat*	Aerial parts	MTT assay, ELISA, annexin V‐FITC/PI staining	Ethanol	MCF‐7	↑ Apoptosis induction, cell cycle arrest	31–299 mg/L	^33^
*Scutellaria baicalensis*	Root	HPLC, staining	Ethanol, ethyl acetate, 1‐butanol, water	MCF‐7	↑ Cytotoxicity ↓ Hypoxic conditions	100 mg/mL	^34^
*Senecio graveolens*	Flower, leaves, stems	Extraction, cytotoxic assays, western blot	Ethanol	ZR‐75‐1, MDA‐MB‐ 231	↑ Cell death, cell cycle arrest ↓ Proliferation	200 μg/mL	^35^
*Withania somnifera*	N/A	Flow cytometry, microarray data analysis, PCR, invasion assay, western blotting	70% ethanol	MDA‐MB‐ 231, MCF‐7	↑ Apoptosis induction, cell cycle arrest	853.6 nM	^36^

## 2 SOURCE OF ENLISTED DIETARY PHYTOCHEMICAL

Phytochemicals are plant‐based compounds founds in vegetables, fruits, beans, grains, and other parts of plants. Bioactive phytochemicals protect cells from cancer‐causing injury.^53^ For instance, daidzein, genistein, epigallocatechin gallate (EGCG), epigallocatechin, and formononetin‐A are phytoestrogen in nature and found in the form of flavonoids in soy and soy products.^54^, ^55^, ^56^, ^57^ Lutein, 3,3‐Diindolylmethane, benzyl isothiocyanate, kaempferol, and quercetin are available in green leafy vegetables including spinach, broccoli, peas, and herbs such as dill, chives, onion, leeks, and egg yolks.^9^, ^58^, ^59^, ^60^ In addition, vegetables such as tomatoes, potatoes, and fruits such as citruses, watermelon, apples, pink guava, pink grapefruit, papaya, passion flower fruit, and dried apricots, are the significant source of 2‐hydroxychalcone,^61^ lycopene,^62^ naringenin.^63^ Also, natural compounds such as nimbolide, sanguinarine, withaferin A, α‐Mangostin, arctigenin, calycosin, curcumin, and flavopiridol are present abundantly in medicinal plants such as *Azadirachta indica* (leaves and seed), *Sanguinaria canadensis* (rhizome), *W. somnifera*, *Tripterygium wilfordix* (roots), *Garcinia mangostana* L.(pericarps), *Arctium lappa* L. (seeds), *Radix astragali* (dry root), *C. longa* (rhizome), and *Dysoxylum binectariferum* (stem and bark), respectively.^36^, ^64^, ^65^, ^66^, ^67^, ^68^ Furthermore, punicalagin, sesamin, shikonin, silibinin, taiwanin A, and wogonin are found in *Punica granatum*, *Cuscuta palaestina* (seed), *Sesamum indicum*, *Lithospermum erythrorhizon* (roots), *Silybum marianum*, *Taiwania cryptomerioides* (bark), *N. sativa* (seeds), and *Anodendron affine* (stems) plants.^69^, ^70^, ^71^, ^72^, ^73^, ^74^ EGCG, and epigallocatechin are known catechin phytochemicals, widely distributed in tea with several health benefits.^75^, ^76^ The source and structure of these phytochemicals are presented in Table 2.

**TABLE 2:** Source and structure of common phytochemicals with anti‐cancer properties.

Compounds	Structure	Source	Ref.
2‐Hydroxychalcone	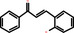	Tomatoes, potatoes, licorice, citruses, apples (*Humulus lupulus* L.)	^61^
3,3‐Diindolylmethane	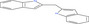	Cruciferous vegetables, that is, brussels sprouts, cauliflower, cabbage, and broccoli	^59^
Apigenin	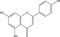	Parsley, chamomile, celery, vine‐spinach, and oregano	^77^
Arctigenin	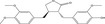	Present in the seeds of *Arctium lappa* L.	^67^
Benzyl isothiocyanate	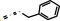	Cruciferous vegetables like 3,3‐diindolylmethane source	^9^
Calycosin	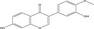	Dry root extract of *Radix astragali*	^68^
Celastrol	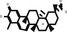	The root extract of *Tripterygium wilfordi* plant	^78^
Coumestrol	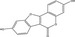	Clover, Kala Chana, Alfalfa sprouts	^79^
Curcumin	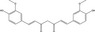	Rhizome of turmeric (*Curcuma longa*)	^80^
Daidzein	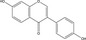	Soybeans and soy products, that is, beans, peas, nuts, coffee, tea, and specific herb like red clover	^54^
EGCG	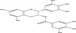	Green tea	^81^
Emodin	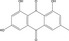	Herbs, that is, *Polygonum cuspidatum*, *Aloe vera*, *Rheum palmatum*, and *Cassia obtusifolia*	^82^
Enterolactone	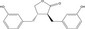	Flaxseed, sesame seed	
Epigallocatechin	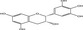	Green tea	^83^
Flavopiridol	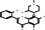	The stem and bark of *Dysoxylum binectariferum* plant	^84^
Formononetin	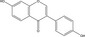	Red clovers, soya bean, milk vetch (*Astragalus mongholicus*)	^55^
Genistein	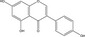	Soybeans and soy products	^56^
Ginsenoside Rh1	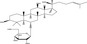	Red ginseng, root	^85^
Ginsenosides	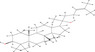	*Panax* species (roots, leaves, stems, flower, fruits)	^86^
Isoliquiritigenin	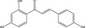	Licorice, extract of *Sinofranchetia chinensis*	^87^
Kempferol	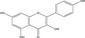	Green leafy vegetables such as broccoli, spinach, and kale, and herbs such as dill, chives, and tarragon, onion, leeks	^60^
Lutein	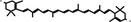	Green leafy vegetables such as broccoli, spinach peas, lettuce, and egg yolks	^58^
Lycopene	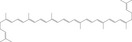	Tomato, watermelon, pink guava, papaya, pink grapefruit, and dried apricots passionflower fruit	^62^
Naringenin	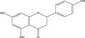	Fruits like citrus species and tomatoes	^63^
Nimbolide	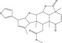	Leaves and flowers of neem (*Azadirachta indica*)	^64^
Pharbilignan C	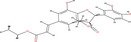	Pharbitidis semen, the seed of morning glory (*Pharbitis nil*)	^88^
Pterostilbene	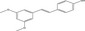	Blueberries, grapes, and tree wood	^89^
Punicalagin	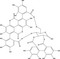	Pomegranate (*Punica granatum*)	^69^
Quercetin	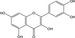	Nuts, apples, onions, olive oil green tea, broccoli, red grapes, dark cherries	^90^
Sanguinarine	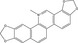	Rhizome of bloodroot (*Sanguinaria canadensis*)	^65^
Withaferin A	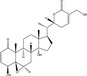	*Withania somnifera*	^36^
α‐Mangostin	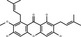	Pericarps of mangosteen	^66^
Resveratrol	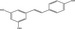	Grapes, peanuts, and soy	^57^
Rg3	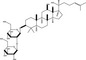	Red ginseng root (*Panax ginseng* C.A. Meyer)	^91^
Rosmarinic acid	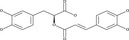	Boraginaceae species and Nepetoideae of the Lamiaceae subfamily	^92^
Sesamin	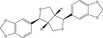	Sesame seeds, *Cuscuta palaestina* plant extract	^48^
Shikonin	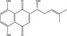	Roots of *Lithospermum erythrorhizon*	^93^
Silibinin	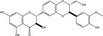	*Silybum marianum* plant	^72^
Sulforaphane	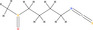	Broccoli, cauliflower, radish, cabbage and arugula	^94^
Taiwanin A	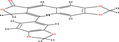	Bark of *Taiwania cryptomerioides*	^73^
Thymoquinone	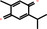	*Nigella sativa* (seeds)	^95^
Wogonin	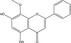	*Scutellaria baicalensis* (dried root), *Scutellaria rivularis, Andrographis paniculata* (wall, leaves)	^74^
Oxymatrine	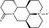	*Sophora flavescens* (quinazine alkaloid extracted)	^96^
Jasmonates	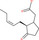	*Camellia sasanqua L*., *Camellia sinensis L*. (anther and pollen)	^97^
Fisetin	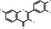	*Fragaria ananassa*, *Malus domestica* (fruit)	^98^

## 3 PHYTOCHEMICALS TARGETING BC CELLS

Therapeutic strategies against BC include surgery chemoradiotherapies, adjuvant/neoadjuvant therapies, hormonal therapies, monoclonal antibodies, immunotherapy, nanomedicines, and small molecular inhibitors.^99^ However, limitations such as resistance, compromised efficacy, and side effects of conventional therapies limit their clinical applications. Thus, plant‐derived anti‐cancer agents with less or no toxic effects can be an alternative chemotherapeutic option. Anti‐cancer activity of phytochemicals is dependent on their multi‐targeted mechanism of action. Since carcinogenesis is a multistep process involving multiple signaling mechanisms, numerous phytochemicals targeting the altered signaling in cancer are considered promising anti‐cancer therapeutics.^100^ Phytochemicals targeting signaling pathways in cancer are summarized (Table 3). The following sections outline the role of potentially bioactive compounds against BC cells with their possible molecular mechanism.

**TABLE 3:** Summary of selected particular phytochemical and their anti‐cancer activity in human breast cancer cell line.

Phytochemical	Dose	Study type	Target	Macular mechanism	Ref.
*Effects of phytochemicals on cell proliferation*					
Formononetin‐A	25 μM	MCF‐7 and MDA‐MB‐231	↓ Tumor growth ↓ Angiogenesis	↓ FGFR2‐mediated Akt signaling	^101^
Sesamin	100 μM	MCF‐7	↓ Proliferation	↓ Cyclin D1 expression	^102^
Curcumin	1.25–5 mg/mL	MDA‐MB‐231 and BT‐483 cell	↓ Proliferation	↓ NF‐κB, and more importantly cyclin D1, CDK4 MMP1 mRNA	^103^
Genistein	40–100 μM	MCF‐7	↓ Proliferation	↓ IGF‐1R‐PI3K/Akt ↓ Bcl‐2/Bax mRNA	45, 104
Lycopene	100 μM	MDA‐MB‐468	↓ Proliferation ↑ Apoptosis	↓ Akt, mTOR ↑ Bax	^105^
Rosmarinic acid	20 μmol/L	MCF‐7	↓ Proliferation	↓ COX‐2 expression, AP‐1 activation, and antagonized the ERK1/2 activation	^106^
Silibinin	50–200 μmol	MCF‐7	↑ Apoptosis ↓ Proliferation	↓ Bcl‐xl ↑ p53, p21, BRCA1, Bak, ATM	^107^
Apigenin	30 μM	MDA‐MB‐468	↓ Proliferation	↑ ROS production ↓ p‐Akt	^108^
Enterolactone	75 μM	MDA‐MB‐231	↓ Proliferation ↓ Migration	↓ PA‐induced plasmin activation ↓ MMP‐2 and MMP‐9	^109^
*Effects of phytochemicals on apoptosis induction*					
Ginsenoside Rh1	50 μM	In vitro MCF‐7, HCC1428	↑ Apoptosis, autophagy	↓ ROS‐mediated PI3K/Akt pathway ↑ ROS production ↑ LC3B and cleaved caspase‐3	^110^
Daidzein	25–100 μM	In vitro MCF‐7	↑ Apoptosis	↑ Bax, cyt c, caspases 9 and 3 ↓ Bcl‐2	^111^
Nimbolide	1.97–5 μM	In vitro MDA‐MB‐231 MCF‐7	Apoptosis autophagy	↓ Bcl2, mTORp62 Beclin 1, LC3B protein	^112^
Lycopene	2–16 μM	In vitro MCF‐7	↓ Proliferation ↑ Apoptosis	↑ p53 and Bax	^113^
Pharbilignan C	5–20 μM	In vitro MDA‐MB‐231	↑ Apoptosis	↑ Bax, caspases 9 and 3 ↓ Bcl‐2	^88^
EGCG	0–80 μM	In vitro T47D	↑ Apoptosis	↓ Telomerase and P13K/AKT ↑ Bax/Bcl‐2, CASP3, CASP9, and PTEN	^83^
Sanguinarine	0–1.5 μM	In vitro MDA‐MB‐231	↑ Apoptosis	↑ ROS generation ↑ cytochrome *c* ↑ caspase‐3 and caspase‐9 ↓ XIAP, cIAP‐1	^114^
Lutein	N/A	In vivo BALB/c mice	↑ Apoptosis ↓ angiogenesis	↑ p53 and Bax ↓ Anti‐apoptotic gene, Bcl‐2	^115^
Kaempferol	20–80 μM	In vitro MCF‐7 cells	↑ Apoptosis	↑ PARP cleavage, Bax ↓ Bcl‐2	^116^
Emodin	40 μM	In vitro Bcap‐37 and ZR‐75‐30	↓ Growth ↑ Apoptosis	↑ Cleaved caspase‐3, PARP, p53 ↑ Bax/Bcl‐2 ratio	^117^
Withaferin A	2.5–5 μM	In vitro MDA‐MB‐231 and MCF‐7	↑ Apoptosis	ROS production, Bax and Bak mitochondrial membrane potential	^118^
Celastrol	1–10 μM	In vitro MDA‐MB‐231 and MCF‐7	Apoptosis	↑ TNF‐α, caspase‐8, caspase‐3, PARP cleavage ↓ Cellular cIAP1 and cIAP2, FLIP, Bcl‐2	^119^
*Effects of phytochemicals on cell cycle regulator*					
Quercetin	5–20 μM	In vitro MCF‐7	↓ Cell cycle progression	↓ Cdc2‐cyclin B1 ↑ p21CIP1/WAF1	^120^
Taiwanin A	5 μg/mL	In vitro MCF‐7	↑ DNA damage ↑ Cell cycle arrest at G(2)/M ↑ Apoptosis	↑ p53, p‐p53, p21, p27	^121^
Coumestrol	50 μM	In vitro MCF‐7	↑ G1/S phase arrest	↑ CDKI p21 and p53	^122^
Ginsenosides	100 μM	In vitro MCF‐7	↓ Proliferation	↓ CDK4, cyclin E2, cyclin D1 ↑ p21WAF1/CIP1, p53 p15INK4B	^123^
Kaempferol	10–6 μM	In vitro MCF‐7	↓ Proliferation ↑ Apoptosis	↓ Capthepsin D, cyclin E and cyclin D1 ↑ Bax and p21	^124^
Thymoquinone	100–200 μM	In vitro MCF‐7, T47D, MDA‐MB231	↓ Proliferation, viability	↓ Cyclin D1, cyclin E, p27, survivin	^125^
Naringenin	0.05–4 μM	In vitro HTB26 and HTB132	↓ Cell growth ↑ Cell cycle arrest at S‐ and G2/M‐phases ↑ Apoptotic cell death ↓ Cell survival factors	↑ p18, p19, p21 ↓ Cdk4, Cdk6, Cdk7, NF‐κB p65	^126^
*Effects of phytochemicals on angiogenesis and metastasis*					
Shikonin	5 μM	In vitro MCF‐7	↓ Migration and invasion	↓ MMP‐9	^127^
Flavopiridol	70 nM	MDA‐MB‐231	↓ Metastasis	↓ MMPs 2 and 9, c‐erbB‐2	^128^
Silymarin	100 μg/mL	MCF‐7 and MDA‐MB‐468	↓ Migration and invasion	↓ VEGF secretion, MMP‐9, AP‐1 activation	^129^
Curcumin	20–100 μM	MCF‐7	↓ Metastasis and migration	↓ uPA, NF‐κB activation	^130^
Arctigenin	10–200 μM	MCF‐7 and MDA‐MB‐231	↓ Cell migration	↓ MMP‐9, urokinase‐type plasminogen activator	^131^
2‐hydroxychalcone and xanthohumol	4.6–18.1 μM	MDA‐MB‐231	↓ Invasive phenotype	↓ MMP‐9 ↓ Bcl‐2	^132^
Enterolactone	25–5 μM	MDA‐MB‐231 cells	↓ Migration and invasion	↓ MMP‐2 and MMP‐9 expressions ↑ MMPs inhibitor	^109^
Quercetin	34 mg/kg	MCF‐7	↓ Angiogenesis	↓ VEGF, VEGFR2, NFATc3, calcineurin pathway	^133^
Rg3	5 mg/kg Rg3, 1 time/2 day	MCF‐7	↓ Invasion and angiogenesis	↓ MMP‐2, MMP‐9, VEGFA, VEGFB, VEGFC, p62, Beclin‐1, P13K, mTOR, Akt and JNK	^134^
Sulforaphane	10 μM	MCF10	↓ Migration, invasion	↓ TNF‐α, MMP‐2, MMP‐9, MMP‐13	^135^
Silibinin	50 μg/mL	MDA‐MB‐468 xenograft model	↓ Metastasis and migration ↓ Tumor volume	↓ EGFR phosphorylation VEGF, MMP‐9, and COX‐2	^136^
Isoliquiritigenin	25–50 μM	MDA‐MB‐231	↓ Migration ↓ Angiogenesis	↓ VEGF, HIF‐1α, MMP‐2, MMP‐9 ↓ p38, Akt, NF‐κB, P13K	^137^
Thymoquinone	100 μL	MCF7 and MDA‐MB‐231	↓ Migration, invasion	↑ TGF‐b, E‐cadherin, cytokeratin 19 ↓ MMP‐2, MMP‐9, Ysnail, Twist, Smad2, NF‐κB	^138^
Punicalagin	N/A	MDA‐MB‐231	↓ Invasion and angiogenesis	↓ VEGF expression ↑ MIF regulation	^139^
*Effects of phytochemicals on hypoxia‐inducible factor*					
EGCG	50–100 mg/kg/day for 4 weeks	In vivo C57BL/6 J mice	↓ Growth ↓ Migration ↓ Angiogenesis ↓ Proliferation	↓ HIF‐1α ↓ NFκB and VEGF expression	^140^
Isoliquiritigenin	25–50 μM	In vitro MDA‐MB‐231	↓ Proliferation	↓ HIF‐1α	^137^
3,3‐Diindolylmethane	50 μM	In vitro MDA‐MB‐231	↓ Angiogenesis	↓ Furin, glucose transporter‐1 ↓ VEGF, enolase‐1 ↓ Phosphofructokinase in hypoxic	^141^
Lyciumbarbarum polysaccharides	0.50 mg/mL	In vitro MCF‐7	↓ Angiogenesis	↓ HIF‐1α mRNA levels	^22^
Wogonin	40 μM	In vitro and vivo MCF‐7, MDA‐MB‐231 Xenograft mouse	↓ Angiogenesis	↑ HIF‐1α degradation ↓ HIF‐1α protein aggregation and translation ↓ Hsp90 client proteins EGFR, Cdk4, and survivin	^142^
*Effects of phytochemicals on mammosphere formation*					
Pterostilbene	25–50 μM	MCF‐7	↓ bCSCs ↓ Mammospheres	↓ CD44, hedgehog, Akt, GSK3b signaling, cyclin D1, c‐Myc ↑ β‐Catenin	^143^
Sulforaphane	50 mg/kg	SUM‐149 and SUM‐159 Y	↓ bCSCs ↓ Mammospheres	↓ NF‐κB p65 subunit, p52	^144^
Benzyl isothiocyanate	3 μmol/g	MDA‐MB‐231, MCF‐7 and SUM159	↓ bCSCs ↓ Mammospheres	↓ Ron, sfRon, ALDH1 ↑ SOX‐2, Nanog, [Oct‐4]	^145^
Resveratrol	100 mg/kg/day	MCF‐7, SUM159	↓ bCSC proliferation ↓ Mammospheres	↓ Wnt, β‐catenin	^146^
Curcumin	5 μM	MCF‐7, MCF10A, SUM149	↓ bCSCs self‐renewal	↓ SCD, CD49f, LDH1A3, TP63	^147^
EGCG	40 μM	MDA‐MB‐231 and MDA‐MB436	↓ bCSCs growth	↓ ER‐a36, MAPK/ERK, EGFR, PI3K/AKT	^148^
*Effects of phytochemicals on inflammation*					
Pomegranate juice	20–80 μmol/L	ApoE‐KO mice J774.A1 macrophage	**↓** Pro‐inflammatory state	**↓** TNF‐α and IL‐6 secretion ↑ IL‐10	^149^
Curcumin	10–20 μM	In vitro MCF‐7	**↓** Inflammation **↓** Cell proliferation ↑ Apoptosis	↑ Blocked the TNF‐α‐induced NF‐κB **↓** Proteasomal activities	^150^
Resveratrol	10 ppm	In vivo Sprague Dawley rats	↑ Cell cycle arrest at S‐G(2)‐M phase **↓** Ductal carcinoma	**↓** NF‐κB, cyclooxygenase‐2, and matrix metalloprotease‐9 expression	^151^
Resveratrol, EGCG, curcumin	–	In vivo Sprague Dawley rats	↑ Pro‐inflammatory mediators in macrophage **↓** Stearic acid‐mediated activation	**↓** TNF‐α, IL‐1β, COX‐2, phospho‐Akt, phospho‐p65, NF‐κB	^152^
*Effects of phytochemicals on enzymatic activity*					
Curcumin	20 μM	In vitro MCF‐7	↓ GSTP1 methylation	↑ Glutathione S‐transferase Pi 1	^153^
Resveratrol	25 μM	In vitro MCF‐7	↑ Enzymatic inhibition	↓ Aromatase mRNA expression ↓ CYP19 promoters activity and II transactivation	^154^
Sulforaphane	25 μM	In vitro MCF10A	Block signaling pathways	↓ COX‐2 expression ↓ ERK1/2‐IKK and NAK‐IKK	^155^
Rosmarinic acid	10 μmol/L	In vitro MCF10A	↓ Pro‐inflammatory gene ↓ Cell proliferation	↑ AP‐1 activation ↓ COX‐2 expression	^106^
Silibinin	200 μM	In vitro MCF‐7 and MDA‐MB231	↓ Cell viability ↓ Tumor inducing genes	↓ COX‐2 expression ↓ TPA‐arbitrated MMP‐9 expression	^156^
Isoliquiritigenin	10–40 μM	In vitro MDA‐MB‐231, BT‐549	↓ Metastasis ↑ Apoptosis	↓ COX‐2, CYP 4A activity ↓ PGE2, PLA2 expression and activity	^157^
Quercetin and epigallocatechin	0.01–500 μM and 0.01–1000 μM	In vitro MCF‐7 and MDA‐MB231	↓ Metabolic process	↓ Glucose uptake ↓ Lactate production	^158^
*Effects of phytochemicals on cell signaling pathways*					
Genistein	100 μM	MCF‐7 and MCF‐7 HER2	Signal inhibition	↓ IκBα, p65 nucleus phosphorylation ↓ NF‐ŚB transcription	^159^
Formononetin	10–100 μM	MCF‐7	↓ Proliferation ↓ Cyclin D1 mRNA expression	↓ IGF1/IGF1R‐PI3K/Akt phosphorylation	^160^
Calycosin	0–100 μM	MCF‐7, T‐47D, MDA‐231 and MDA‐435	↓ Growth and induce apoptosis	↓ IGF‐1R, MAPK, (PI3K)/Akt pathways	^161^
Arctigenin	200 μM	MCF‐7, MDA‐MB‐231	↓ Metastasis	↓ Akt, NF‐κB phosphorylation ↓ MAPK (ERK 1/2 and JNK 1/2) signaling	.^131^
Resveratrol	100 mg/kg	MCF‐7	↑ Autophagy ↑ Cytotoxicity	Wnt/β‐catenin	^146^
Apigenin	50 μM	MCF‐7/HER2 and MCF‐7 vec	↑ Apoptosis ↓ Proliferation	↓ p‐JAK1, p‐STAT3, NF‐κB, p‐IκBa	^162^
Silibinin	50 μM	MDA‐MB‐231	↑ Apoptosis	↓ ERK, Akt, Notch‐1	^163^
Pterostilbene	0–100 μM	MDA‐MB‐468	↑ Apoptosis ↓ Proliferation	↑ ERK1/2 ↓ p21, YAkt, mTOR	^164^
Naringenin	250 μM	MCF‐7	↑ Apoptosis ↓ Proliferation	↓ P13K, MAPK, ERK1/2, AKT	^165^
α‐Mangostin	30 μM	T47D, MDA‐MB‐468, SKBR3, and AU565	↑ Apoptosis ↓ Proliferation	↓ P13K, ERK1/2, ERa, Akt, ERK1/2, MAPK ↑ p‐p38, p‐JNK1/2	^166^
*Effects of phytochemicals on epigenetic regulator*					
Genistein	80 μM	In vitro MDA‐MB‐231	↑ Epigenetic stability	↑ p21^WAF1^ (p21) and p16^INK4a^ ↓ BMI1 and c‐MYC expression	^167^
Lycopene	3.125 μM, 2 μm/week)	In vitro MCF‐7 and MDA‐MB‐468, MCF10A	↑ Epigenetics stability	↑ Demethylases the GSTP1, RARbeta2 and the HIN‐1 genes	^168^
Curcumin	40 μM	In vitro MDA‐MB‐361	↓ Cell growth ↑ Repression tumor suppressor gene	↓ Sp1 expression, DLC1 methylation	^169^
EGCG	15 μM	In vitro MCF7 and MDA MB 231	↓ Cell viability	↓ RARb2, cyclin D2 methylation, TMS1 methylation, MGMT methylation	^170^
Sulforaphane	5 μM	In vitro MDA‐MB‐231	↑ Cell death ↓ Proliferation	↓ HDAC demethylation	^171^

### 3.1 Inhibition of cell proliferation

Cellular proliferation is essential for all multicellular organisms to develop bodies and organs during embryogenesis. However, in the case of cancer, abnormal cell proliferation is due to changing the expression or activity of protein associated with cell proliferation or cell cycle regulation. Phytochemicals and their derivatives can inhibit the growth and expansion of BC cells by targeting cell cycle regulatory proteins.^172^ For example, the naturally active compound formononetin (25 μΜ) suppresses tumor growth and angiogenesis in MCF‐7 and MDA‐MB‐231 tumor models by targeting the FGFR2‐mediated Akt signaling pathway.^101^ Treatment of MCF‐7 cells by silibinin (50–200 μmol) prevented cell proliferation through modulating the expression of apoptosis‐related proteins such as Bcl‐xl, bak, p53, p21,^107^ whereas sesamin (100 μM) could inhibit MCF‐7 cell proliferation by down‐regulating cyclin D1 expression.^102^ Curcumin mediated its anti‐proliferative activity against BC (MDA‐MB‐231 and BT‐483) cells by regulating the expression of NF‐κB, cyclin D1, CDK4, and MMP1.^103^ Chen et al. noted that Genistein (40–100 μM) exhibited anti‐proliferative activity by deactivating the IGF‐1R‐PI3K/Akt signaling pathway along with increasing Bax/Bcl‐2 expressions in MCF‐7 cells,^104^ whereas lycopene showed similar activities by increasing Bax expression without changing Bcl‐xL in MDA‐MB‐468 cancer cells.^105^ Scheckel KA reported that the anti‐proliferative activity of rosmarinic acid (20 μmol/L) is associated with a decrease in COX‐2 expression and activation of AP‐1 and ERK1/2 in MCF‐7 cells.^106^ Harrison et al. reported that apigenin arrests the cell cycle at the G2/M phase, followed by down‐regulation p‐Akt in MDA‐MB‐468 cancer cells.^108^ Furthermore, enterolactone (ENL) has been shown to suppress cell proliferation by lowering uPA‐mediated plasmin activation and down‐regulation of MMP‐2 and MMP‐9 in MDA‐MB‐231 cells.^109^ Therefore, phytochemicals could act as potent inhibitors of cell proliferation in BC cells by suppressing cell survival signaling, cell cycle regulatory protein, and regulating apoptosis‐related proteins.

### 3.2 Apoptosis inductions

Apoptosis, a programmed cell death mechanism, plays a crucial role in cancer pathogenesis and maintenance by regulating cell death and survival based on specific signals.^173^ Apoptosis can be executed via two mechanisms, that is, the extrinsic and intrinsic mitochondrial pathways.^174^ Both of these pathways are regulated through several regulatory proteins.^175^ The extrinsic pathway, for instance, is associated with the Fas ligand, Fas‐associated protein with death domain initiator pro‐caspase‐8, and many caspases contributing to the cascade amplification.^176^ In contrast, the intrinsic pathway involves apoptosis‐related proteins such as Bax, Bak, Bcl2, Cyto‐c, adaptor protein Apaf‐1, and active caspases.^177^ Thus, regulating these proteins by phytochemicals could be an alternative for better management of patients with BC. Ginsenoside Rh1 (50 μM, 24 h) exerted a potential anti‐cancer effect against BC (MCF‐7 and HCC1428) cells through induction of apoptosis and autophagy.^110^ Nimbolide (1.97–5 μM) and pharbilignan C (5–20 μM) are associated with the down‐regulation of Bcl‐2/Bax along with up‐regulation of caspases (caspases 9 and 3), thereby leading to induced apoptosis of MDA‐MB 231 and MCF‐7 cells through mitochondrial‐dependent intrinsic pathways.^88^, ^112^ Furthermore, nimbolide induces cancer cell autophagy by inhibiting mammalian target of rapamycin (mTOR) and p62 expression and increasing two essential proteins, Beclin 1, and LC3B expression.^112^ Jin et al. reported that daidzein (25–100 μM) treatment of MCF‐7 BC cells caused up‐regulation of Bax protein and down‐regulation of Bcl‐2 protein expression, leading to cytochrome *c* release, which in turn induced apoptosis via activating caspases‐9 and 7.^111^ Choi et al. reported that treatment of BC cells (MDA‐MB 231) with sanguinarine (0–1.5 μM) caused apoptosis by generating ROS, leading to the transfer of cytochrome‐*c* into cytosol followed by caspase‐3 and caspase‐9 activation and inactivation of anti‐apoptosis factor XIAP and cIAP‐1.^114^ Chew et al. noted that lutein regulated the apoptosis pathway by increasing tumor suppressors (and apoptosis genes) such as p53 and Bax and decreasing anti‐apoptosis genes such as *Bcl‐2* expression in female BALB/c mice.^115^ Zu et al. reported that emodin (40 μM) inhibits growth by inducing apoptosis through up‐regulating cleaved Bax/Bcl2_,_ p53, caspase‐3, PARP cleavage in human BC (ZR‐75‐30 and Bcap‐37) cells.^117^ Another phytochemical, withaferin A (2.5–5 μM) induced apoptosis through ROS production by modulating the expression of Bax/Bak in MDA‐MB 231 and MCF‐7 BC cells.^118^ Furthermore, Mi et al. reported that celastrol (1–10 μM) induced apoptosis by modulating the expression of TNF‐α, caspase‐8, caspase‐3, and PARP cleavage along with inhibition of anti‐apoptotic proteins such as cellular cIAP1 and cIAP2, FLIP, and Bcl‐2 expression in MCF‐7 and MDA‐MB 231 cells.^119^ Also, lycopene and EGCG induced apoptosis by up‐regulating the expression of p53 and Bax/Bcl‐2 ratio with down‐regulating telomerase and P13K/AKT in MCF‐7 and T47D cancer cells.^83^, ^113^ Furthermore, curcumin and resveratrol can induce apoptosis through the regulation of Bax/Bcl2, whereas thymoquinone, apigenin, pterostilbene, and sulforaphane are associated with apoptosis by regulating caspases cascade and signal transduction mechanism in multiple human BC cells.^144^, ^164^, ^178^, ^179^, ^180^, ^181^ Therefore, phytochemicals inhibit BC progression by apoptosis induction, which mediates either intrinsic or extrinsic, and sometimes both pathways.

### 3.3 Inducing cell cycle arrest

The cell cycle is a principal physiological mechanism regulating tissue homeostasis and development in multicellular organisms. Therefore, alterations in the cell cycle cause cancer. Thus, novel strategies have been developed targeting altered cell cycles or components. Checkpoints in the cell cycle arrest cell cycle progression in the case of DNA damage, allowing time for DNA repair.^182^, ^183^ In numerous breast carcinomas, phytochemicals inhibit the passage of the cell cycle by modulating checkpoints components such as lowering cyclins (D1 and E) levels and cyclin‐dependent CDKs etc., and by up‐regulating the expression of proteins such as CDK inhibitors (p21 and p27). For example, quercetin halts the cell cycle at the G2/M phase by raising Cdk‐inhibitor, especially p21CIP1/WAF1 and its associated protein Cdc2‐cyclin B1 complex in MCF‐7 cancer cells.^120^ Treatment of coumestrol (50 μM) caused cell cycle arrest at the G1/S phase, followed by upregulations of regulatory protein CDKI and p21 and p53 in MCF‐7 cells.^122^ Also, taiwanin A treatment was associated with the up‐regulation of p21, p27, p53, and p‐p53 in MCF‐7 cells in a dose‐dependent manner.^121^ Kim et al. reported that ginsenosides (100 μM) had arrested the cell cycle at G0/G1 phase via inhibiting Cyclin D1, Cyclin E2, and their associated enzyme CDK4, along with up‐regulating p15INK4B, p21WAF1/CIP1 and p55 level in MCF‐7 cells.^123^ Another phytochemical, kaempferol, reduced MCF‐7 cell growth by down‐regulating cathepsin D, cyclin E, and cyclin D1 expressions and up‐regulating Bax and p21.^124^ Furthermore, thymoquinone (100–200 μM) significantly inhibited the expression of cyclin D1 and E, resulting in promoting the survival of multiple BC (MCF‐7, T47D, and MDA‐MB‐231) cells.^125^ Moreover, naringenin is an essential plant chemical that can regulate cell cycle checkpoints by suppressing CDK4, CDK6, and CDK7 with up‐regulating p18, p19, and p21 in BC (HTB26 and HTB132) cells.^126^ Altogether, phytochemicals halt the progression of the cell cycle of BC cells by either inhibiting the expression and activity of cyclins (B1, D1, and E) and CDKs (4, 6, 7) or increasing the expression of CDKs inhibitors (p18, p21, p27, and p53).

### 3.4 Inhibition of angiogenesis and metastasis

Angiogenesis is closely associated with metastasis. These processes are acquired at a critical density of arteries and occur as the tumors expand, spread, or become less differentiated.^184^ Growth factors (VEGF, PDGF, FGF, and EGF), matrix metalloproteinase (MMP‐2, MMP‐9), intracellular adhesion molecules‐1(ICAM‐1), etc., are associated with these processes. Thus, they can be a potential target for cancer therapeutics development. It was reported that phytochemicals have significant anti‐metastatic and anti‐angiogenesis effects by inhibiting MMP‐9 and MMP‐2 and suppressing VEGFR‐2 expression, thereby inhibiting the growth and invasiveness and adhesion of cancer cells.^185^, ^186^ Flavopiridol, a phytochemical (70nM ), inhibited secretion of metalloproteinase, especially MMPs (MMP 2 and 9) and c‐erbB‐2 in MDA‐MB‐231 cells, which is associated with the reduction of cell invasion inhibition.^128^ Nobel phytochemicals such as 2‐hydroxy chalcone and xanthohumol exerted potent inhibitory effects on the invasive phenotype of MDA‐MB‐231 cells by inhibiting MMP‐9 expression with Bcl‐2 down‐regulation and shikonin showed a similar result in MCF‐7 cells.^127^, ^132^ The reduced level of MMP‐9 and urokinase‐type plasminogen activator was observed in MDA‐MB‐231, TPA‐induced MCF‐7 cells followed by a lower dose of arctigenin (10–200 μM) treatment in turn inhibited cells' movement.^131^ Similarly, plant‐derived silymarin decreased VEGF secretion, blocked PMA‐induced inhibition of MMP‐9, and blocked AP‐1 activation, thus, modulating MAP signaling in MCF‐7 and MDA‐MB‐ 468 cells in a dose‐dependent manner.^129^ In addition, it could downregulate VEGF activity in MDA‐MB‐231 cells, inhibiting angiogenesis.^139^ Mali et al. reported that ENL (2–25 μM) could downregulate MMP‐2 and MMP‐9 activity while up‐regulating tissue inhibitors, that is, metalloproteinases 1 and 2 (TIMP‐1 and TIMP‐2), in MDA‐MB‐231 cells.^109^ Another phytochemical Rg3 (5 mg/kg/2 day) suppressed cell migration and angiogenesis while promoting autophagy through decreasing angiogenesis factors (VEGFA, VEGFB, VEGFC), metastatic factors (MMP‐2, MMP‐9), signaling molecules (P13K, Akt, mTOR, JNK, p62, and Beclin‐1) in MCF‐7 cells.^134^ Treatment with quercetin (34 mg/kg) inhibits angiogenesis by reducing the activity of VEGF, VEGFR2, and NFATc3 in human BC xenografted nude mice. Also, it defeats calcineurin activity and its mediated pathway.^133^ Kil et al. reported that silibinin (50 μg/mL) could inhibit metastasis and migration by inhibiting EGFR phosphorylation and suppressing VEGF, MMP‐9, and COX‐2 in MDA‐MB‐468 cells, resulting in decreased tumor volume in the triple‐negative BC xenograft model.^136^ Isoliquiritigenin (25–50 μM) treatment inhibited signaling molecules such as NF‐κB, P13K/Akt, and p38, decreasing MMP‐2, MMP‐9, VEGF, and HIF‐1α expressions leading to reduce the motility of MDA‐MB‐231 cancer cells.^137^ Another phytochemical, thymoquinone, could modulate the expression of epithelial markers such as E‐cadherin, cytokeratin 19, and mesenchymal markers such as MMP‐2, MMP‐9, integrin‐aV, TGF‐b in MCF‐7 and MDA‐MB‐231 cells.^138^ Thus, the suppression of angiogenesis and metastasis in BC cells can be achieved by treating with plant products or plant‐derived bioactive compounds, which could suppress matrix metalloproteinases, growth factor expressions, and signaling mechanisms (Figure 3).

### 3.5 Inhibition of hypoxia‐inducible factor

Tumor hypoxia refers to cells being deprived of normal oxygen due to low oxygen levels in the tumor microenvironment. Hypoxia induces multiple signaling cascades such as MAPK, phosphatidyl‐inositol 3‐kinase (PI3K), HIF, and NF‐κB pathways in cancer cells, leading to feedback loops of both positive and negative, and enhancing or diminishing hypoxic effects.^187^ It was also found that hypoxia regulates several cellular phenomena, such as the expression of drug efflux proteins, apoptosis, DNA damage, the efficiency of chemotherapy, angiogenesis, and metastasis.^187^ Therefore, targeting hypoxia‐inducible factor 1 (HIF‐1), a crucial component of hypoxia, could be a potential strategy against hypoxia‐induced cancer cell growth and progression. Several phytochemicals can directly inhibit HIF‐1‐related genes, including *GLUT‐1*, *CDKN1A*, and *VEGF*. This inhibition ultimately results in a decrease in tumor angiogenesis, migration, and chemotaxis. According to Wang et al. isoliquiritigenin (25–50 μM) treatment suppressed P13K/Akt, NF‐κB signaling pathways via modulating the expression of VEGF, HIF‐1α, and MMP‐2, MMP‐9 expressions, leading to limit the migration of MDA‐MB‐231 cells.^137^ Riby et al. demonstrated that 3,3‐diindolylmethane (50 μM) exhibited anti‐cancer activity by decreasing the expression of hypoxia‐responsive factors such as furin, and glucose transporter‐1, VEGF, enolase‐1, and phosphofructokinase in hypoxic specific MDA‐MB‐231 cells.^141^ In addition, lyciumbarbarum polysaccharides inhibit HIF‐1α protein aggregation by altering mRNA levels and VEGF mRNA expression leading to inhibit the nuclear translocation of HIF‐1α in MCF‐7 cells.^142^ Another study showed that EGCG (50 μg/mL) inhibits breast tumor formation, proliferation, migration, and angiogenesis by inhibiting HIF‐1α in MCF‐7 and MDA‐MB‐231 cells.^140^ Wang et al. noted that shikonin (10 μM) suppresses the expression of HIF‐1α in MDA‐MB‐231 cells in hypoxic conditions.^188^ Thus, phytochemicals inhibit cancer progression by regulating hypoxia‐inducible factors by aggregation or degradation (Figure 3).

### 3.6 Inhibition of oxidative stress and redox signaling

Reactive oxygen species (ROS) such as hydroxyl radical, superoxide anion radical, hydrogen peroxide, oxygen singlet, nitric oxide radical, and peroxynitrite extreme play essential roles in the initiation and development of tumors.^189^ These species contribute to harmful genomic material, making them genetically unstable. Also, they act as intercessors in mitogenic and survival signaling using adhesion molecules and receptors of growth factors. Enzymes involved in an antioxidant system, such as catalase (CAT), superoxide dismutase (SOD), peroxiredoxins (PRXs), glutathione peroxidase (GPX) and glutathione reductase, are essential for maintaining cellular redox system.^190^ However, it is not easy to mitigate the excessive production of ROS by cellular antioxidant enzymes.^191^ It was noted that phytochemicals could modulate oxidative stress and redox signaling by regulating the expression of these enzymes. For example, Singh et al. reported protective roles of resveratrol via increasing Nrf‐2 expression, which could up‐regulate the expression of antioxidant genes such as SOD3, NQO1, and 8‐oxoguanine DNA glycosylase 1 (OGG1).^192^ In addition, biochanin A (500 μg/g) has shown anti‐cancer activity in oxidative stress‐mediated cancer by up‐regulating CAT, DT‐diaphorase, GST, GPx, and SOD, along with the reduction of lipid peroxidation and lactate dehydrogenase activities significantly.^193^ Nadal‐Serrano et al. reported the protective effects of Genistein on oxidative stress, redox signaling, and mitochondria, followed by up‐regulation of ERβ in T47D BC cells.^194^ Moreover, Fan et al. reported that 3,3′‐diindolylmethane (1 μmol/L) protects BC cells against oxidative stress by stimulating the expression of nuclear factor erythroid 2 in BC cells.^195^ Therefore, phytochemicals regulate oxidative‐mediated cancer progression by controlling potent oxidative markers, including Nrf‐2 expression and antioxidant gene expression in both in vitro and in vivo models.

### 3.7 Inhibition of mammosphere formation

The formation of the mammosphere is an essential characteristic of cancer progression, mainly cancer stem cells (CSCs). Several studies reported that BC cells, including non‐adherent, non‐differentiating CSC, form the mammosphere.^196^ CSCs are believed to be associated with cancer reappearance, metastasis, and resistance to anti‐cancer drugs. Thus, targeting breast CSCs by inhibiting mammosphere formation can be an alternative approach for managing BC. Naturally occurring plant‐based compounds can prevent cancer cells and CSCs by decreasing mammosphere formation.^197^ For example, Wu et al. demonstrated that pterostilbene suppressed mammosphere formation BCSCs growth by reducing CD44^+^ surface antigen expression and stimulating β‐catenin phosphorylation.^143^ The pterostilbene also modulates the hedgehog/Akt/GSK3b signaling pathway via the down‐regulation of cyclin D1 with c‐Myc expression.^143^ Another phytochemical, sulforaphane (SFN), reduced the number and size of ALDH1‐positive (BCSC) cells, resulting in the inhibition of mammospheres formation in both in vitro and in vivo models.^198^ In addition, SFN‐pretreated ALDH^+^ cells showed enhanced sensitivity to taxane, thereby blocking mammospheres formation significantly.^144^ Fu et al. noted that resveratrol (100 mg/kg/day) treatment against BCSCs induces autophagy by suppressing the Wnt/β‐catenin signaling pathway in MCF‐7 and SUM159 cells.^146^ Colacino et al. found that curcumin downregulates the expression of CD49f, ALDH1A3, PROM1, and TP6 in MCF‐7, MCF10A, SUM149‐derived stem cells' growth and proliferation.^147^ Benzyl isothiocyanate (3 μmol BITC/g) treatment suppressed the expression of both Ron and sfRon in cultured MCF‐7 derived stem cells and tumor xenografts, indicating that benzyl isothiocyanate treatment caused inhibition bCSCs in vitro and in vivo.^145^ Piperine (10 μM) significantly decreased mammosphere formations in stem cells derived from BC.^199^ Therefore, phytochemicals showed anti‐cancer activities by inhibiting mammosphere formation in multiple breast carcinomas by suppressing signaling pathways or their components (Figure 3).

### 3.8 Inhibition of inflammation

Inflammation is a biological reaction to cellular injury produced due to infections, chronic irritation, and other inflammatory responses.^200^ Information suggests that inflammatory cells, including neutrophils, macrophages, dendritic cells, eosinophils, and lymphocytes were associated with tumor formation, development, angiogenesis, and progression.^201^, ^202^ Interestingly, significant research demonstrated that natural compounds prevent inflammation by regulating antioxidant defence mechanisms via modulating Phase I, and Phase II enzymes or inflammatory cells or factors in cancer.^203^ An in vitro study reported the therapeutic advantage of polyphenols on the inflammatory phenotype of macrophages.^149^ In this study, supplemented pomegranate juice polyphenols reduced M1‐macrophages mediated pro‐inflammatory stimulation in the J774.A1 macrophage‐like cells in a dose‐depended manner.^149^ Curcumin also exhibited anti‐cancer properties against inflammation‐associated carcinogenesis by inhibiting TNF‐α mediated NF‐κB activation and inhibiting the proteasomal activity of IκB kinase in MCF‐7 cells.^150^ Synergistically, using Sprague Dawley rats, curcumin with resveratrol inhibits inflammation by lowering NF‐κB and reducing inflammatory markers such as COX‐2 and MMP‐8 expression animal model.^151^ In addition, Dharmappa et al. reported that genistein had anti‐inflammatory properties in cancer by inhibiting sPLA activity in a concentration‐dependent manner.^204^ Furthermore, multiple dietary polyphenols combination from zyflamend, (e.g., resveratrol, curcumin, and EGCG), decreased the expression of pro‐inflammatory markers such as COX‐2, IL‐1β, TNF‐α, phospho‐Akt, phosphor‐p65, and NF‐κB‐binding activity in C57BL/6J female mouse model.^152^ Therefore, natural phytochemicals are potent oncogenic inhibitors by regulating inflammation through regulating TNF‐α mediated NF‐κB, IκB kinase, COX‐2 and MMP‐8, IL‐1β, TNF‐α, phospho‐Akt, phosphor‐p65, and NF‐κB‐binding activity in numerous cancer models.

### 3.9 Enzymatic inhibition

Interfering the enzymatic functionality associated with cancer pathogenesis potentially prevents BC development. Phytochemical treatment could inhibit Phase I enzymes, inducible nitric oxide synthase, cyclooxygenase‐2, xanthine oxide, aromatase, and many more in cancer.^205^ Supplementation of curcumin (20 μM) is associated with reversing hypermethylation of the Glutathione S‐Transferase Pi 1 (GSTP1) gene, resulting in reactivation via modulation of epigenetics mechanism in MCF‐7 cells.^153^ It is also reported that curcumin (35 μM) inhibited MCF‐7 cell proliferation by Nrf2 arbitrated Flap endonuclease‐1 (Fen1) expression,^206^ whereas resveratrol (25 mM) inactivates the aromatase enzyme by removing the CYP19 promoters I.3 and II transactivation.^154^ Furthermore, resveratrol regulates other cancer‐associated enzymes such as COX‐2, NQO‐2, and GSTP 1.^207^ In addition, Barbara E reported that cabbage juice inhibits BC (MCF10 and MDA‐MB‐231) cells by inhibiting aromatase expression.^208^ Similarly, rosmarinic acid (10 μM) acts as an essential COX‐2 inhibitor through AP‐1 activation in MCF‐7 cells in a dose‐dependent manner.^106^ Furthermore, another natural product, isoliquiritigenin (10–40 μM), showed chemopreventive actions by targeting metabolic enzymes such as COXs, PLA2s, LOXs, and PGE2, cytochrome P450 4 (CYP 4A) activity in MDA‐MB‐231, BT‐549 BC cells.^157^ Quercetin and epigallocatechin could decrease glucose consumption and lactate production in MCF‐7 and MDA‐MB231 cells, inhibiting cancer‐related metabolic pathways.^158^ Thus, phytochemicals showed anti‐cancer efficacy through regulation of enzymatic functions, that is, by regulating estrogen synthesizing enzymes such aromatase, estrogen metabolizing enzymes CYP 4A, CYP19 suppressing COX‐2 expression, or regulating GSTP1 in BC cells. Therefore, natural phytochemicals are potent oncogenic inhibitors by regulating several enzymes, including hypermethylation of the GSTP1, Flap endonuclease‐1, aromatase expression, CYP19 promoters I.3 and II transactivation, and numerous enzymes in different cell lines.

### 3.10 Natural compounds targeting cell signaling pathways

mTOR, PI3K, protein kinase B (Akt), MAPK/ERK, Wnt, Notch, and hedgehog signaling pathways are associated with the regulation of cell proliferation, differentiation, survival, apoptosis, invasion, migration, angiogenesis, and metastatic spread of cancer cells.^209^, ^210^ Phytochemicals elicit anti‐cancer actions by regulating these pathways or components.^159^ For example, Seo et al. reported that genistein (100 μM) inhibited IκBα phosphorylation and maintained its association with p65–p50 heterodimer, which blocked their nuclear translocations, and p65 phosphorylation, which in turn prevented the transcription of NF‐κB targeted genes.^159^ Also, genistein inhibited MAPK signaling by suppressing MEK5, ERK5, and p‐ERK5 levels in MDA‐MB231 cells,^211^ whereas apigenin inhibited ERK 1/2 and JNK 1/2 phosphorylation via inhibiting MAPK signaling in MCF‐7 cells.^131^ Calycosin and formononetin, two phytochemicals, regulated PI3K/Akt pathways through IGF‐1R protein expression along with the inhibition of Akt phosphorylation in T47D and MCF‐7 cells.^160^, ^161^ In addition, Fu et al. reported that resveratrol (100 mg/kg) down‐regulates Wnt/β‐catenin signaling, inducing autophagy in MCF‐7 cells^146^ and inhibiting cell proliferation of SKBR‐3 BC cells through down‐regulation of various signaling pathways such as p‐Akt, PI3K, Akt, mTOR.^212^ Apigenin inhibited MCF‐7 cells by inducing apoptosis by inhibiting NF‐κB, STAT3, and p53 signaling.^162^ Silibinin is associated with the death of MDA‐MB‐231 cells by regulating Notch‐1 signaling pathways.^163^ Pterostilbene regulates ERK1/2 activation, decreased cyclin D1, p‐AKT, mTOR, and increased p21, Bax protein, but not Bcl‐xL.^164^ Hatkevich showed that naringenin inhibits PI3K, thus disrupting proliferation signaling in MCF‐7 cells through ERK1/2, AKT, and MAPK signaling pathways,^165^ whereas α‐Mangostin mediated its anti‐tumor effect through decreasing HER2, Akt, and P13K along with increasing p‐p38 and p‐JNK1/2 phosphorylation.^166^ Therefore, phytochemicals inhibit NF‐κB, PI3K/Akt, MAPK/ERK, p‐mTOR, Wnt, Notch‐1, and hedgehog signaling pathways by modulating their components or upkeep/downstream molecules in BCs (Figure 4).

### 3.11 Natural compounds targeting epigenetic control

Accumulating information suggests that previous studies have shown that phytochemicals can modulate the epigenetics of cancer cells by regulating the methylation of DNA via DNA methyltransferase activity and histone modifications, resulting in inhibiting the oncogenic miRNA expression and increasing tumor‐suppressing miRNA expression.^213^, ^214^, ^215^ Studies have shown that genistein could inhibit primary breast carcinogenesis by increasing some tumor suppressor protein i,e and p16, p16 (INK4a), p21, p21 (WAF1) expression, along with decreasing expression oncogene, that is, BMI1, and c‐MYC in estrogen negative MDA‐MB‐231 cell line.^167^ Moreover, genistein attributed its anti‐cancer activity in BC cells by demethylating and reactivating methylation‐silenced tumor suppressor genes via direct contact with inhibition of both DNA methyltransferase 1 (DNMT1) catalytic domain activation and DNMT1 expression.^213^ Furthermore, genistein decreased the oncogenic miR‐155 expression with increasing expression of miR‐155 targets such as Forkhead box O3 and casein kinase, p27, phosphatase, and tensin homolog (PTEN), which in turn that promote apoptosis and antiproliferation of MDA‐MB‐435 cells.^162^, ^216^ Lycopene up‐regulated glutathione S‐transferase pi gene (GSTP1) expression and demethylases the GSTP1 in MCF‐7, MDA‐MB‐468 cells, whereas induced RARbeta2 and HIN‐1 genes demethylation in BC (MCF10A) cells in a dose‐dependent manner.^168^ Similarly, SFN (5 μM) significantly inhibits HDAC through demethylation in MDA‐MB‐231 cells.^171^ Liu et al. reported that curcumin activated the promoter of deleted in liver cancer 1 by suppressing methylation status, with the help of down‐regulating the Sp1 transcription factor in MDA‐MB‐361 cells.^169^ Also, EGCG (15 μM) treatment is associated with epigenetic changes that can increase DNMTs transcripts expressions such as DNMT1, DNMT3a, and DNMT3b in both MCF‐7 and MDA‐MB‐361 cells.^170^ Thus, phytochemicals have the potential to modulate the epigenetic make‐up of BC cells via regulating DNA methylation and histone modification; therefore, they could control the expression of oncogenes and tumor suppression genes in BC cells. The summary of phytochemicals that act against epigenetics regulation is summarized in Figure 2.

### 3.12 Natural compounds targeting the immune system

**FIGURE 1:**
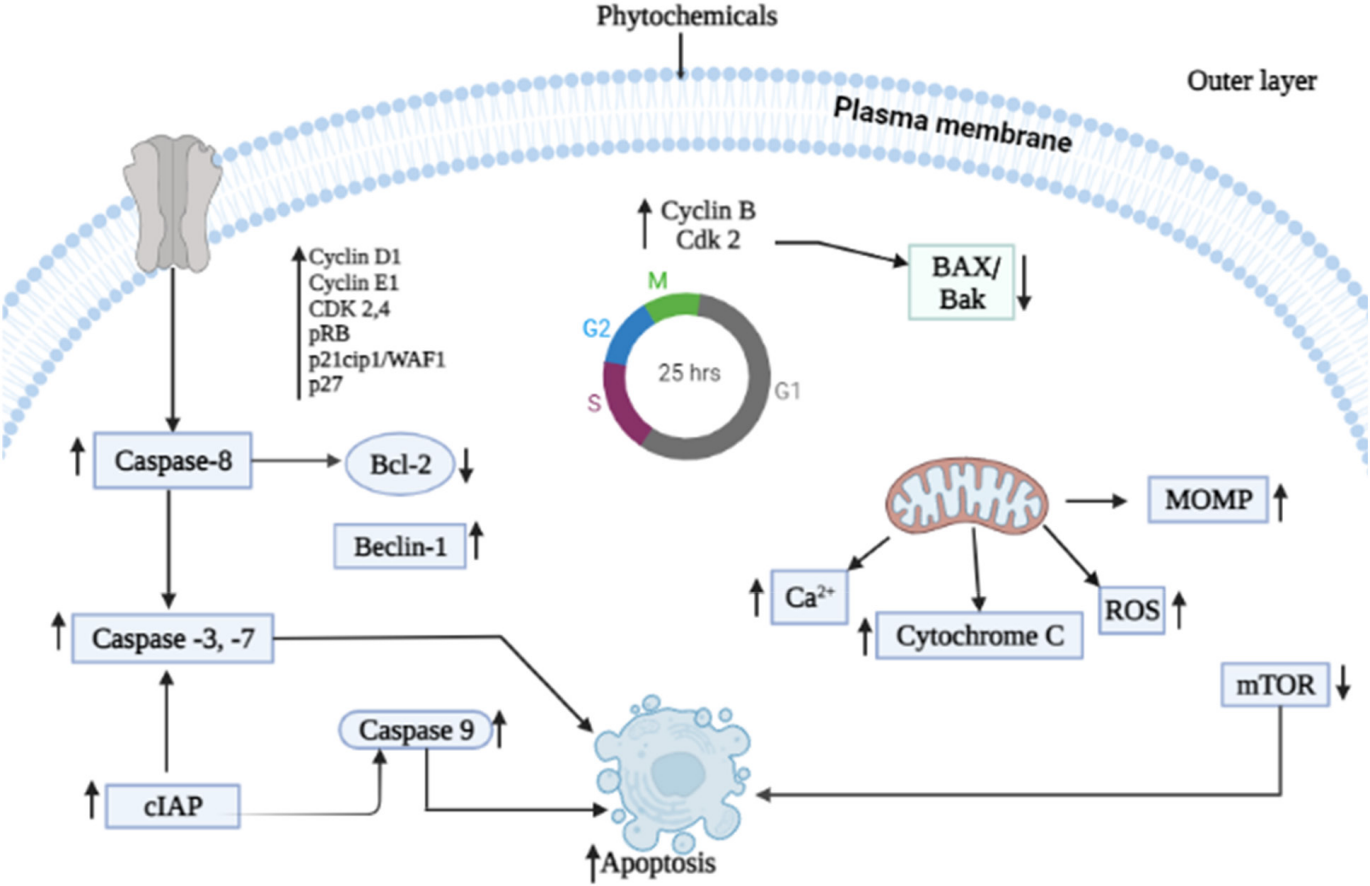
Breast cancer management by dietary phytochemicals through apoptosis and cell cycle: Phytochemicals activate caspase‐8 through modulating TRAIL‐ and FAS‐associated receptors. Activated caspase‐8 mediated activation of some effector caspase‐3 and caspase‐7 attributed to the extrinsic pathway of apoptosis. Moreover, the anti‐apoptotic protein BCL2 mediates activation of BAK, BAX. These powerful mechanisms increase cytosolic Ca^2+^, cytochrome *c*, and reactive oxygen species (ROS). Cytochrome *c* sequentially activates caspase‐9, which is simultaneously activated by effector caspase‐3 and caspase‐7 attribute to apoptosis. Activation of tumor suppressor protein (p21CIP1/W, p27, p53, pRB, and AF1) and suppression of cyclin (cyclin B, D1, E1) with associating enzymes (CDK 2, 4) by phytochemicals regulated cell cycle and cell proliferation.

**FIGURE 2:**
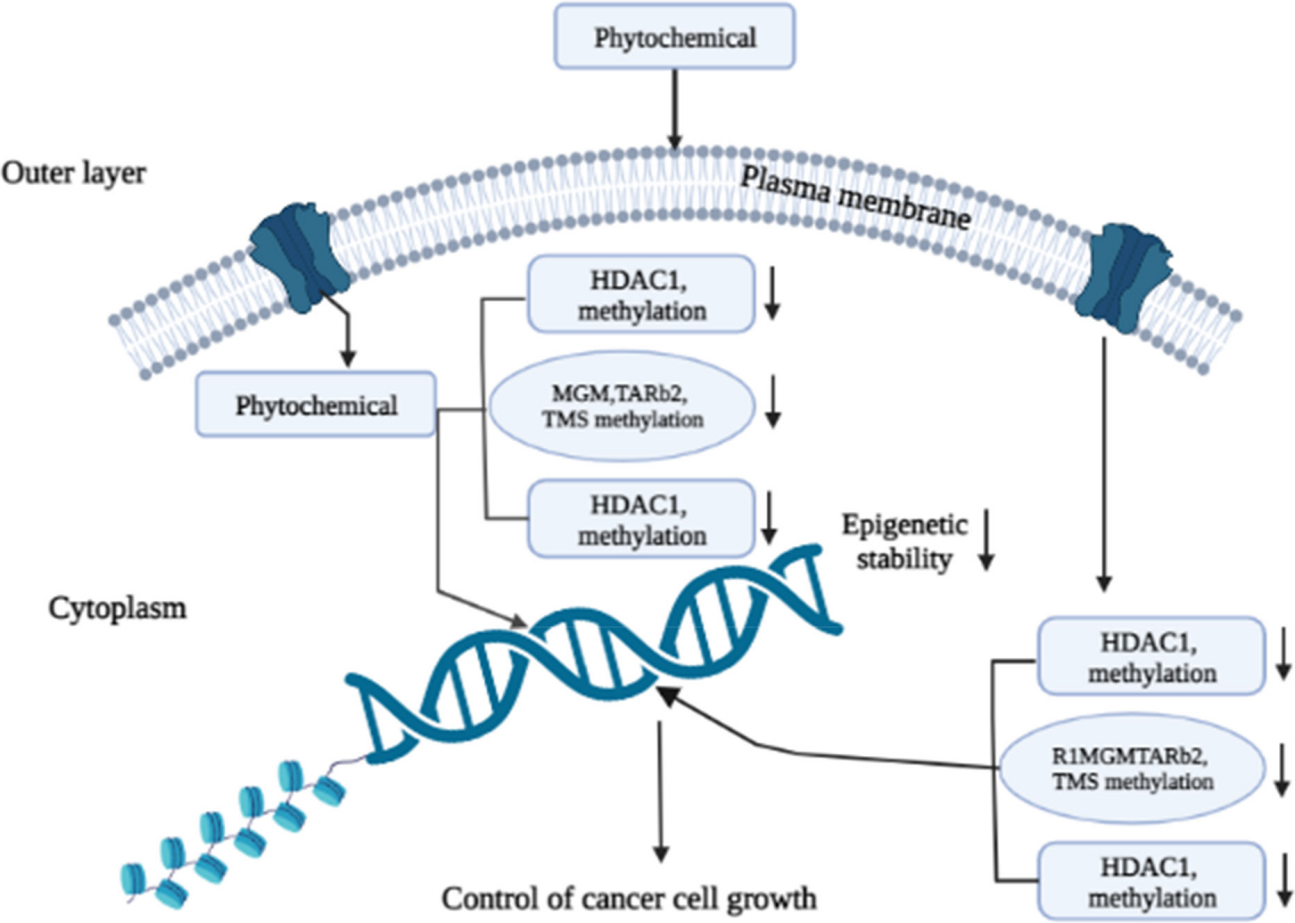
Breast cancer management by dietary phytochemicals through enzymatic control of epigenetics factors: Breast cancer can regulate epigenetics factors. The key epigenetic regulatory protein R1MGMTARb2, TMS methylation, BMI1, c‐MYC, HDAC1 methylation, histone modification can be regulated by dietary phytochemicals; leading to show anti‐cancer effect.

**FIGURE 3:**
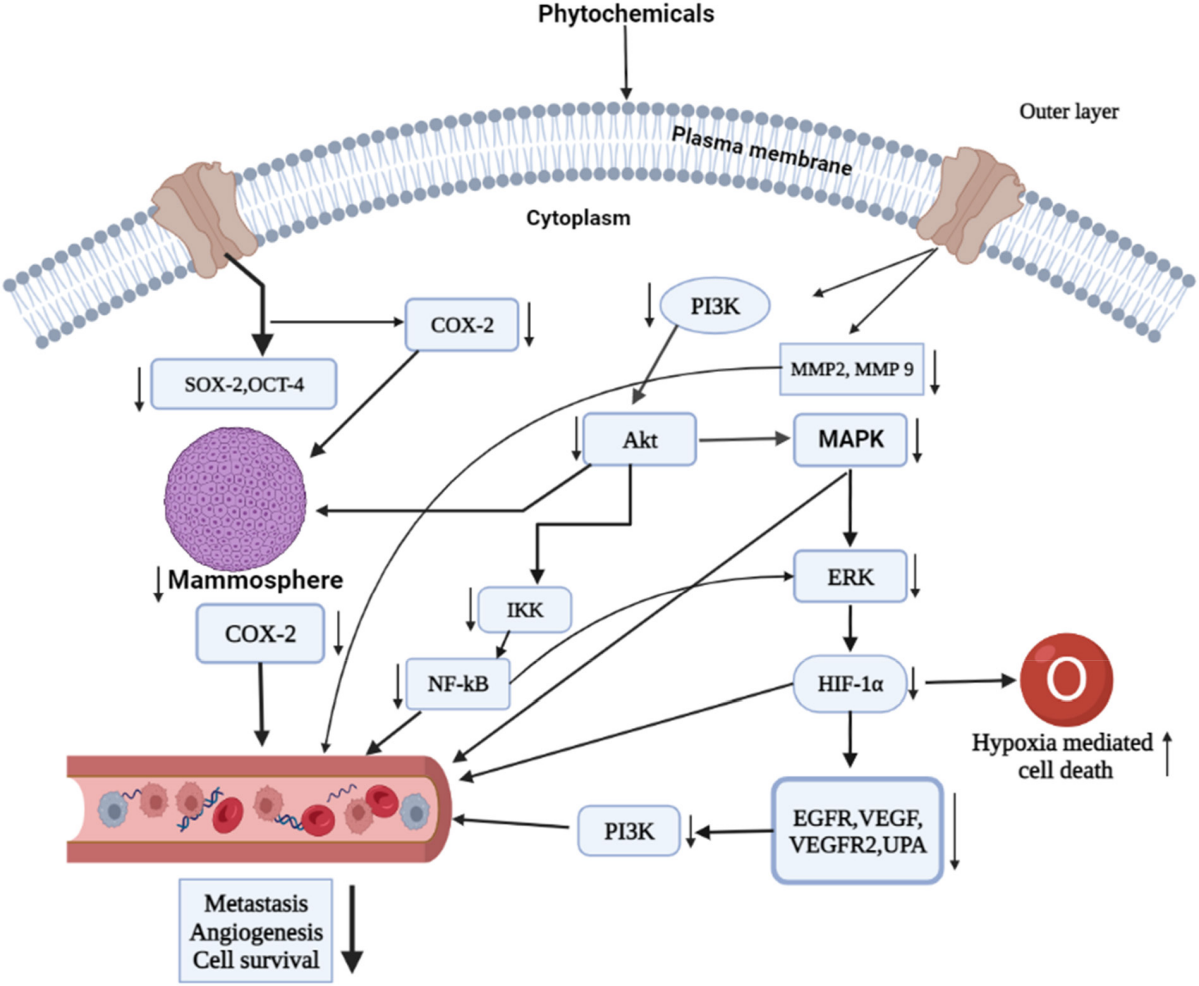
Control of breast cancer by dietary phytochemicals targeting multiple patways: Targeting the multiple signal transduction, phytochemicals can suppress some cell signaling pathways, that is, PI3k/Akt/mTOR, MAPK/ERK, NF‐κB, HIF‐1α, leading to a decrease cancer cell metastasis, angiogenesis, and survival. Followed by the signal transductions, phytochemicals can mitigate important metastatic and angiogenic factors including EGFR, VEGF, VEGFR2, NF‐κB, MMP2, MMP9, COX‐2, and ERK in breast cancer cell line.

**FIGURE 4:**
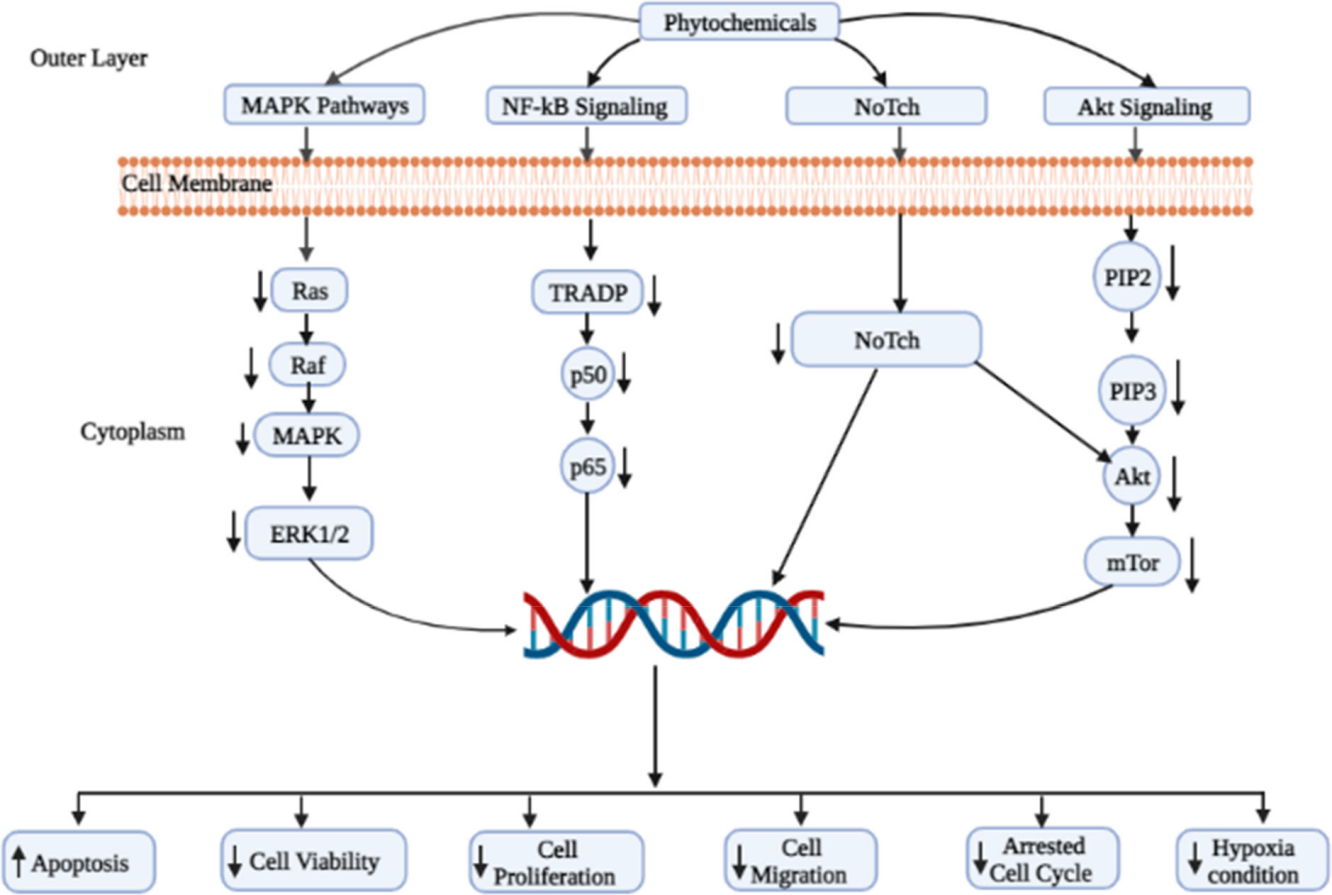
Phytochemicals targeted signaling pathways associated with breast cancer treatment: The schematic diagram represents the overview of molecular mechanisms of phytochemicals mediated inhibition of breast cancer cell growth through the Notch, MAPK, NF‐κB, and Akt pathways.

Phytochemicals include substances found in nature that can be bioactive and possess an immune system‐stimulating effect.^217^ For example, curcumin, a clinically naturally occurring compound, has immunomodulatory properties that suppress PHA‐induced T cell proliferation, IL‐2, NO, and NF‐κB while increasing NK cell cytotoxicity in mouse macrophage cells RAW.264.7.^218^ A study involving C57BL/6 mice found that apigenin may influence the alteration of dendritic cells and other immune cell functions.^219^ Daidzein, has a modulatory function on nonspecific immunity in Swiss mice when given in high doses since it enhances the phagocytic response of peritoneal macrophages.^220^ Additionally, in male Kunming mice exposed to 60Coγ radiation, EGCG significantly reduced immune system destruction by inducing macrophage phagocytosis, boosting the activity of the antioxidant enzymes, that is, SOD and GSH‐Px (glutathione peroxidase), raising glutathione level, and preventing lipid peroxidation.^221^ Conversly, genistein regulates immunological response in female Sprague Dawley, promoting IL‐4 synthesis while inhibiting IFN‐γ release and balancing Th1/Th2 cells.^222^ Furthermore, kaempferol had immune‐suppressive effects on cold‐stressed, 6‐7‐week‐old SPF mice, decreasing the levels of activated pro‐inflammatory cytokines like IL‐9 and IL‐13, CD8^+^ T cells and raising anti‐inflammatory cytokines and CD4^+^ T cells.^223^ Therefore, selected phytochemicals have the potential to activate immune system including numerous immune cells including NK cell, CD8^+^ T, CD4^+^ T and cytokines like IL‐9 and IL‐13 to fight against BC cells. A summary of the anti‐cancer mechanism of phytochemicals in BC treatment is presented in Table 3 and Figures 1, 2, 3, 4.

## 4 THE ABILITY OF PHYTOCHEMICALS TO ALLEVIATE THE RESISTANCE OF ANTI‐CANCER DRUGS

Due to numerous significant challenges, such as multi‐drug resistance, treating cancer patients is becoming more difficult.^224^ Drug efflux, drug inactivation, drug detoxification, drug target modification, involvement of CSCs, miRNA dysregulation, epigenetic alteration, and other numerous irregular DNA damage/repair mechanisms, tumor microenvironment, and ROS modulation are just a few potential defensive processes that could result in this resistance mechanism.^40^, ^225^, ^226^ P glycoprotein (P‐GP), MRP 1, MRP 1–9, BCRP, and changes in beta‐tubulin are a few proteins that are connected to drug resistance in cancer.^227^ The multi‐drug resistance protein P‐glycoprotein (P‐gp) is overexpressed in the membrane of cancer cells, where it commonly increases drug efflux and contributes to the emergence of treatment resistance in malignancies.^228^ Hence, inhibiting MDR‐efflux proteins may help improve cancer therapy's effectiveness. For example, Biochanin A exhibits this type of action. Soo et al. demonstrated that Biochanin A treatment increased [3H]‐DNM accumulation by reducing DNM efflux and caused MDR to be reversed by suppressing P‐gp activity in MCF‐7/ADR BC cells.^225^ The effects of phloretin on P‐gp activity were examined (HTB26) by measuring the uptake of rhodamine 123 in a variety of cancer cells, including human MDR1 gene‐transfected mouse lymphoma cells (L1210) and human BC cells MDA‐MB‐231 expressing the MRP1 pump.^226^ Genistein indirectly raises intracellular drug concentration, including doxorubicin concentration, but does not directly alter P‐gp activity in a BC cell lines. In a study, Castro and Altenberg reported that genistein reduced the photo‐affinity labeling of P‐gp with [3H] azidopine, a P‐gp substrate, indicating that genistein might suppress rhodamine123 efflux in human MCF‐7 cells by directly interacting with P‐gp to impede P‐gp‐mediated drug efflux.^229^ The other component that stimulates the formation of BC is human epidermal growth factor receptor 2 (HER2), a tyrosine kinase (TK) receptor that belongs to the EGFR family. Curcumin was reported to have the capacity to alter the EGFR signaling pathway, which is linked to the growth, differentiation, adhesion, and migration of cancer cells.^230^, ^231^ According to Chandrika et al. hesperetin at 10‐500 μM promotes apoptosis in MDA‐MB‐231 and SKBR3 BC cells and inhibits their ability to proliferate. Dietary flavonoid hesperetin reduces the development of MDA‐MB‐231 BC cells by inhibiting the activity of HER2 Tyrosine Kinase (HER2‐TK), causing MMP loss, chromatin condensation, and activating caspase‐8 and‐3, which causes cell cycle arrest at the G2 phase.^232^ Sesamin inhibited cell migration at the same dosage and cells by delaying the G1 phase and down‐regulating PDL‐1, MMP‐9, and MMP‐2. Sesamin's ability to inhibit cell proliferation was demonstrated by Yokota et al. in BC cells. They discovered that sesamin inhibited growth at doses of 1–100 M by increasing retinoblastoma protein dephosphorylation and decreasing cyclin D1 gene expression, which mediates cyclin D1 degradation.^102^ The co‐treatment of resistant (MCF‐7R) cells with Apigenin, which reduced MDR1 expression at the mRNA and protein levels in both resistant and non‐resistant cells, significantly reduced DOX resistance in the MCF‐7 cell line. In both the MCF‐7 and MCF‐7R cell lines, apigenin strongly inhibited the phosphorylation and activation of the JAK2 and STAT3 proteins.^233^ By lowering Bcl‐2, Nimbolide induces the expression of the proteins Bax and caspases with a modulation of the expression of HDAC‐2 and H3K27Ac, and stopping the progression of the cell cycle, as well as reduced the growth of MDA‐MB‐231 and MCF‐7 cells. Increasing Beclin 1 and LC3B and decreasing p62 and mTOR protein expression in BC cells. Nimbolide also activated autophagy signaling.^112^ Combining Sanguinarine with TRAIL therapy may break BC cells' resistance caused by overexpression of Akt or Bcl‐2. In human BC MDA‐231 cells, Sanguinarine triggered apoptosis, which resulted in decreased pro‐caspase‐3, Bcl‐2, cIAP2, XIAP, and c‐FLIPs protein levels and increased ROS production.^234^ When Emodin was applied to the BC cells Bcap‐37 and ZR‐75‐30, it was shown to suppress proliferation, induce apoptosis, and decrease Bcl‐2 while increasing levels of cleaved caspase‐3, PARP, p53, and Bax.^117^ In MCF‐7 and MDA‐MB‐231 cells, Isoliquiritigenin lowered cell survival and clonogenic potential, triggered apoptosis, suppressed mRNA expression of many AA‐metabolizing enzymes, including PLA2, COX‐2, and CYP‐4A, and reduced production of PGE2 and 20‐HETE. Moreover, it reduced the expression of phospho‐PI3K, phospho‐PDK, phospho‐Akt, phospho‐Bad, and Bcl‐xL, triggering caspase cascades that ultimately led to the cleavage of PARP.^235^ The expression pattern of β‐catenin in BC tissue are high than the normal tissue. EGCG thus decreased the viability of MDA‐MB‐231 cells by lowering the levels of β‐catenin, cyclin D1, and p‐AKT. Moreover, pretreatment of MDA‐MB‐231 cells with PI3 kinase inhibitors, such wortmannin or LY294002, enhanced the suppressive effect of EGCG, given after 24 h, on the production of β‐catenin.^236^ By transfecting the plasmid and inducing cytotoxicity and autophagy in BCSCs derived from MCF‐7 and SUM159, Resveratrol inhibits the Wnt/β‐catenin signaling pathway and excessive production of the β‐catenin protein.^146^ The impact of Wogonin supplementation on cell survival and proliferation has been shown to be effective against a variety of BC cell lines, including TNBC and its related cell lines, BT‐549 and MDA‐MB‐231. Additionally, wogonin inhibits the cell cycle of cancer cell lines by inhibiting the expression of cyclin D1, cyclin B1, and CDK1, inducing apoptosis, improving the Bax/Bcl‐2 ratio, and increasing caspase‐3 cleavage.^237^ In ER‐positive BC cells like MCF‐7 and T‐47D cells, Calycosin tends to suppress proliferation and trigger apoptosis. This effect is caused by ER‐induced inhibition of IGF‐1R as well as the targeted control of the MAPK and (PI3K)/Akt pathways.^161^

## 5 LIMITATIONS AND PROSPECTS OF PHYTOCHEMICALS IN BREAST CANCER THERAPY DEVELOPMENT

Several factors interfere with the conventional therapeutic options used to treat BC. Phytochemicals offer a broad spectrum of pharmacological effects, which might benefit the clinical management of patients with BC. Phytochemicals are an effective therapeutic agent due to their several biological properties. Though phytochemicals have enormous benefits, there are significant constraints in achieving the actual effectiveness of phytochemicals‐based therapeutic for the management of patients with BC due to the lack of systematic and proper information in this field. In addition, to develop a clinically useful drug, a series of preclinical and clinical it must pass in vitro, in vivo, and clinical trials (Phase I–IV) studies must be accomplished with clinical benefit. Furthermore, long‐term studies are still required to determine therapeutic interactions, in vivo pharmacokinetic attributes, effective doses, suitable administration routes, and defined mass and/or nanoformulation of these phytochemicals. To estimate bioactivities, the structure–activity relationship must be established. Gathering additional information regarding phytochemicals' synergistic actions when combined with other phytochemicals, it is possible to boost their activity and prevent the anti‐cancer profile by modifying conventional medications. Moreover, these phytochemicals could be used in computational chemistry research, such as docking, neural networking, and pharmacophore‐based virtual screening programs for the drug development sector. Therefore, these phytochemicals could potentially become a potent chemotherapeutic anti‐cancerous substance in managing BCs, at least at the cellular level and could be formulated for clinical applications if all of the strategies are accomplished.

## 6 CONCLUSION

Although the complete molecular mechanisms for BC pathogenesis are yet to be established, whereas the mortality rates associated with this cancer are still rising worldwide. Thus, developing an effective therapeutic, especially from natural resources, that is, phytochemical‐based therapeutic, could provide significant clinical benefit in the management of patients with BC. The details mechanism of anti‐cancer activity from in vitro, preclinical and clinical studies suggested that phytochemicals mediate their anti‐cancer efficacy through targeting apoptosis proteins, including anti‐apoptotic proteins (Bcl‐2) and apoptotic proteins (Bax, Bak, Bad, and Caspase), arresting cell cycle and proliferation. They modulate the expression of growth‐related genes, for instance, inhibiting expression and activity of cyclins (B1, D1, E) and CDKs (4, 6, 7) or increasing the expression of CDKs inhibitors (p18, p21, p27, and p53). Inhibits metastasis and angiogenesis by controlling the expression of MMP‐2,8 and 9, Wnt/‐catenin, PARP, oxidative markers, including Nrf‐2, antioxidant‐related gene, inhibiting mammosphere formation, regulating inflammation via modulating TNF‐α, NF‐κB, IκB kinase, COX‐2, IL‐1β, TNF‐α, phospho‐Akt, phospho‐p65. Also, regulation enzymatic functions (i.e., aromatase, estrogen metabolizing enzymes CYP 4A, CYP19 suppressing COX‐2 expression, or regulating GSTP1), targeting cell signaling (NF‐κB, PI3K/Akt, MAPK/ERK, p‐mTOR, Wnt, Notch‐1, hedgehog), epigenetics control (regulating DNA methylation and histone modification), activate immune system (NK cell, CD8^+^ T, CD4^+^ T, cytokines like IL‐9 and IL‐13) in BC cell lines.

To conclude, phytochemicals may be used as an alternative and complementary therapeutic option in BC treatments due to their therapeutic benefits. However, further studies are needed to conduct before taking phytochemicals as a food supplement to manage and prevent BC until clinically proven standard drugs are not available in pharma‐markets.

## AUTHOR CONTRIBUTIONS

**Md Sohel:** Conceptualization (supporting); data curation (lead); resources (lead); validation (lead); visualization (supporting); writing—original draft (lead); writing—review and editing (supporting). **Suraiya Aktar:** Resources (supporting); writing—original draft (supporting). **Partha Biswas:** Resources (supporting); visualization (supporting). **Md. Al Amin:** Resources (supporting); writing—original draft (supporting). **Md. Arju Hossain:** Data curation (supporting); resources (supporting). **Nasim Ahmed:** Resources (supporting); writing—original draft (supporting). **Md. Imrul Hasan Mim:** Visualization (supporting). **Farhadul Islam:** Supervision (supporting); validation (supporting); writing—review and editing (supporting). **Md. Abdullah Al Mamun:** Conceptualization (lead); resources (supporting); supervision (lead); validation (supporting); visualization (lead); writing—original draft (supporting); writing—review and editing (lead).

## FUNDING INFORMATION

This research did not receive any specific grant from funding agencies in the public, commercial, or not‐for‐profit sectors.

## CONFLICT OF INTEREST STATEMENT

The authors have declared that there are no conflicts of interest.

## Data Availability

Data included in article/supplementary material/referenced in article.

## References

[1] Sun YS , Zhao Z , Yang ZN , et al. Risk factors and preventions of breast cancer. Int J Biol Sci. 2017;13:1387‐1397. 29209143 10.7150/ijbs.21635 PMC5715522

[2] Karpuz M , Silindir‐Gunay M , Ozer AY . Current and future approaches for effective cancer imaging and treatment. Cancer Biother Radiopharm. 2018;33:39‐51. 29634415 10.1089/cbr.2017.2378

[3] Nobili S , Lippi D , Witort E , et al. Natural compounds for cancer treatment and prevention. Pharmacol Res. 2009;59:365‐378. 19429468 10.1016/j.phrs.2009.01.017

[4] Rodriguez EB , Flavier ME , Rodriguez‐Amaya DB , Amaya‐Farfán J . Phytochemicals and functional foods. Current situation and prospect for developing countries. Segur Aliment Nutr. 2015;13:1‐22. doi:10.20396/san.v13i1.1841

[5] Cragg GM , Grothaus PG , Newman DJ . Impact of natural products on developing new anti‐cancer agents. Chem Rev. 2009;109:3012‐3043. 19422222 10.1021/cr900019j

[6] Sharma SB , Gupta R . Drug development from natural resource: a systematic approach. Min Rev Med Chem. 2015;15:52‐57. 10.2174/138955751501150224160518 25986040

[7] Wang R , Li YLH , et al. Antitumor activity of the *Ailanthus altissima* bark phytochemical ailanthone against breast cancer MCF‐7 cells. Oncol Lett. 2018;15:6022‐6028. 29552229 10.3892/ol.2018.8039 PMC5840722

[8] Singh RK , Ranjan A , Srivastava AK , et al. Cytotoxic and apoptotic inducing activity of *Amoora rohituka* leaf extracts in human breast cancer cells. J Ayurveda Integr Med. 2020;11:383‐390. 30846274 10.1016/j.jaim.2018.12.005 PMC7772503

[9] Nakamura Y , Yoshimoto M , Murata Y , et al. Papaya seed represents a rich source of biologically active isothiocyanate. J Agric Food Chem. 2007;55:4407‐4413. 17469845 10.1021/jf070159w

[10] Shahruzaman SH , Mustafa MF , Ramli S , Maniam S , Fakurazi S , Maniam S . The cytotoxic properties of *Baeckea frutescens* branches extracts in eliminating breast cancer cells. Evid Based Complement Alternat Med. 2019;2019:1‐9. 10.1155/2019/9607590 PMC6507242 31178918

[11] Benarba B , Meddah B , Aoues A . *Bryonia dioica* aqueous extract induces apoptosis through mitochondrial intrinsic pathway in BL41 Burkitt's lymphoma cells. J Ethnopharmacol. 2012;141:510‐516. 22465729 10.1016/j.jep.2012.02.052

[12] Kushwaha PP , Vardhan PS , Kapewangolo P , et al. Bulbine frutescens phytochemical inhibits notch signaling pathway and induces apoptosis in triple negative and luminal breast cancer cells. Life Sci. 2019;234:116783. 31442552 10.1016/j.lfs.2019.116783

[13] Kaur V , Kumar M , Kumar A , Kaur S . *Butea monosperma* (Lam.) Taub. bark fractions protect against free radicals and induce apoptosis in MCF‐7 breast cancer cells via cell‐cycle arrest and ROS‐mediated pathway. Drug Chem Toxicol. 2020;43:398‐408. 30293450 10.1080/01480545.2018.1497051

[14] Huyen CTT , Luyen BTT , Khan GJ , et al. Chemical constituents from *Cimicifuga dahurica* and their anti‐proliferative effects on MCF‐7 breast cancer cells. Molecules. 2018;23:1083. 29734650 10.3390/molecules23051083 PMC6102574

[15] Estanislao Gómez CC , Aquino Carreño A , Pérez Ishiwara DG , et al. *Decatropis bicolor* (Zucc.) Radlk essential oil induces apoptosis of the MDA‐MB‐231 breast cancer cell line. BMC Complement Altern Med. 2016;16:266. 27491777 10.1186/s12906-016-1136-7 PMC4974778

[16] Beier BA . A revision of the desert shrub *Fagonia* (Zygophyllaceae). Syst Biodivers. 2005;3:221‐263.

[17] Li P , AnandhiSenthilkumar H , Wu S‐b , et al. Comparative UPLC‐QTOF‐MS‐based metabolomics and bioactivities analyses of *Garcinia oblongifolia* . J Chromatogr B Analyt Technol Biomed Life Sci. 2016;1011:179‐195. 10.1016/j.jchromb.2015.12.061 26773895

[18] Jiang F , Li Y , Mu J , et al. Glabridin inhibits cancer stem cell‐like properties of human breast cancer cells: an epigenetic regulation of miR‐148a/SMAd2 signaling. Mol Carcinog. 2016;55:929‐940. 25980823 10.1002/mc.22333

[19] Liu Z , Liu M , Liu M , Li J . Methylanthraquinone from *Hedyotis diffusa* WILLD induces Ca^2+^‐mediated apoptosis in human breast cancer cells. Toxicol In Vitro. 2010;24:142‐147. 19686834 10.1016/j.tiv.2009.08.002

[20] Barani M , Mirzaei M , Torkzadeh‐Mahani M , Nematollahi MH . Lawsone‐loaded niosome and its antitumor activity in MCF‐7 breast cancer cell line: a nano‐herbal treatment for cancer. DARU. 2018;26:11‐17. 30159762 10.1007/s40199-018-0207-3 PMC6154483

[21] Yerlikaya S , Baloglu MC , Diuzheva A , Jekő J , Cziáky Z , Zengin G . Investigation of chemical profile, biological properties of *Lotus corniculatus* L. extracts and their apoptotic‐autophagic effects on breast cancer cells. J Pharm Biomed Anal. 2019;174:286‐299. 31185340 10.1016/j.jpba.2019.05.068

[22] Huang X , Zhang QY , Jiang QY , Kang XM , Zhao L . Polysaccharides derived from *Lycium barbarum* suppress IGF‐1‐induced angiogenesis via PI3K/HIF‐1α/VEGF signalling pathways in MCF‐7 cells. Food Chem. 2012;131:1479‐1484.

[23] Sun J , Rui HL . Apple phytochemical extracts inhibit proliferation of estrogen‐dependent and estrogen‐independent human breast cancer cells through cell cycle modulation. J Agric Food Chem. 2008;56:11661‐11667. 19053381 10.1021/jf8021223

[24] Chon SU , Kim YM , Park YJ , Heo BG , Park YS , Gorinstein S . Antioxidant and antiproliferative effects of methanol extracts from raw and fermented parts of mulberry plant (*Morus alba* L.). Eur Food Res Technol. 2009;230:231‐237.

[25] Rohini B , Akther T , Waseem M , Khan J , Kashif M , Hemalatha S . AgNPs from *Nigella sativa* control breast cancer: an in vitro study. J Environ Pathol Toxicol Oncol. 2019;38:185‐194. 31679281 10.1615/JEnvironPatholToxicolOncol.2019027318

[26] Yu JS , Kim AK . Platycodin D induces apoptosis in MCF‐7 human breast cancer cells. J Med Food. 2010;13:298‐305. 20412017 10.1089/jmf.2009.1226

[27] Elmaidomy AH , Mohyeldin MM , Ibrahim MM , et al. Acylated iridoids and rhamnopyranoses from *Premna odorata* (Lamiaceae) as novel mesenchymal–epithelial transition factor receptor inhibitors for the control of breast cancer. Phytother Res. 2017;31:1546‐1556. 28809058 10.1002/ptr.5882 PMC5628144

[28] Sartippour MR , Seeram NP , Heber D , et al. Rabdosia rubescens inhibits breast cancer growth and angiogenesis. Int J Oncol. 2005;26:121‐127. 15586232

[29] Basso AV , Leiva González S , Barboza GE , et al. Phytochemical study of the genus *Salpichroa* (Solanaceae), chemotaxonomic considerations, and biological evaluation in prostate and breast cancer cells. Chem Biodivers. 2017;14:e1700118. 10.1002/cbdv.201700118 28581196

[30] Noori S , Hassan ZM , Mohammadi M , Habibi Z , Sohrabi N , Bayanolhagh S . Sclareol modulates the Treg intra‐tumoral infiltrated cell and inhibits tumor growth in vivo. Cell Immunol. 2010;263:148‐153. 20409537 10.1016/j.cellimm.2010.02.009

[31] Abu‐Dahab R , Afifi F , Kasabri V , Majdalawi L , Naffa R . Comparison of the antiproliferative activity of crude ethanol extracts of nine salvia species grown in Jordan against breast cancer cell line models. Pharmacogn Mag. 2012;8:319‐324. 24082637 10.4103/0973-1296.103664 PMC3785171

[32] Xu X , Rajamanicham V , Xu S , et al. Schisandrin a inhibits triple negative breast cancer cells by regulating Wnt/ER stress signaling pathway. Biomed Pharmacother. 2019;115:108922. 31048190 10.1016/j.biopha.2019.108922

[33] Azadmehr A , Hajiaghaee R , Baradaran B , Haghdoost‐Yazdi H . Apoptosis cell death effect of *Scrophularia variegata* on breast cancer cells via mitochondrial intrinsic pathway. Adv Pharm Bull. 2015;5:443‐446. 26504768 10.15171/apb.2015.060 PMC4616888

[34] Wang CZ , Li XL , Wang QF , Mehendale SR , Yuan CS . Selective fraction of *Scutellaria baicalensis* and its chemopreventive effects on MCF‐7 human breast cancer cells. Phytomedicine. 2010;17:63‐68. 19836937 10.1016/j.phymed.2009.07.003 PMC2789205

[35] Echiburú‐Chau C , Alfaro‐Lira S , Brown N , et al. The selective cytotoxicity elicited by phytochemical extract from *Senecio graveolens* (Asteraceae) on breast cancer cells is enhanced by hypoxia. Int J Oncol. 2014;44:1357‐1364. 24535330 10.3892/ijo.2014.2302

[36] Xiao X , Wu ZC , Chou KC . A multi‐label classifier for predicting the subcellular localization of gram‐negative bacterial proteins with both single and multiple sites. PLoS ONE. 2011;6:e20592. doi:10.1371/journal.pone.0020592 21698097 PMC3117797

[37] Yousif MG , Al‐Mayahi MH . Phylogenetic characterization of staphylococcus aureus isolated from the women breast abscess in Al‐Qadisiyah governorate, Iraq. J Pharm Sci Res. 2019;11:1001‐1005.

[38] Watkins R , Wu L , Zhang C , Davis RM , Xu B . Natural product‐based nanomedicine: recent advances and issues. Int J Nanomedicine. 2015;10:6055‐6074. 26451111 10.2147/IJN.S92162 PMC4592057

[39] Vemuri SK , Banala RR , Subbaiah GPV , Srivastava SK , Reddy AVG , Malarvili T . Anti‐cancer potential of a mix of natural extracts of turmeric, ginger and garlic: a cell‐based study. Egypt J Basic Appl Sci. 2017;4:332‐344.

[40] Sohel M , Sultana H , Sultana T , et al. Chemotherapeutic potential of hesperetin for cancer treatment, with mechanistic insights: a comprehensive review. Heliyon. 2022;8:1‐15. 10.1016/j.heliyon.2022.e08815 PMC8810372 35128104

[41] Roy M , Mukherjee S , Sarkar R , Biswas J . Curcumin sensitizes chemotherapeutic drugs via modulation of PKC, telomerase, NF‐κB and HDAC in breast cancer. Ther Deliv. 2011;2:1275‐1293. 22826883 10.4155/tde.11.97

[42] Fan XJ , Wang Y , Wang L , Zhu M . Salidroside induces apoptosis and autophagy in human colorectal cancer cells through inhibition of PI3K/Akt/mTOR pathway. Oncol Rep. 2016;36:3559‐3567. 27748934 10.3892/or.2016.5138

[43] Das M , Kandimalla R , Gogoi B , et al. Mahanine, a dietary phytochemical, represses mammary tumor burden in rat and inhibits subtype regardless breast cancer progression through suppressing self‐renewal of breast cancer stem cells. Pharmacol Res. 2019;146:104330. 31251988 10.1016/j.phrs.2019.104330

[44] Paul P , Biswas P , Dey D , et al. Exhaustive plant profile of “*Dimocarpus longan* lour” with significant phytomedicinal properties: a literature based‐review. Processes. 2021;9:1803. doi:10.3390/pr9101803

[45] Sohel M , Biswas P , Al Amin M , et al. Genistein, a potential phytochemical against breast cancer treatment‐insight into the molecular mechanisms. Processes. 2022;10:415. doi:10.3390/pr10020415

[46] Biswas P , Dey D , Biswas PK , et al. A comprehensive analysis and anti‐cancer activities of quercetin in ROS‐mediated cancer and cancer stem cells. Int J Mol Sci. 2022;23:11746. 36233051 10.3390/ijms231911746 PMC9569933

[47] Dey D , Hasan MM , Biswas P , et al. Investigating the anticancer potential of salvicine as a modulator of topoisomerase II and ROS signaling cascade. Front Oncol. 2022;12:899009 35719997 10.3389/fonc.2022.899009 PMC9198638

[48] Sohel M , Islam MN , Hossain MA , et al. Pharmacological properties to pharmacological insight of sesamin in breast cancer treatment: a literature‐based review study. Int J Breast Cancer. 2022;2022:1‐13. doi:10.1155/2022/2599689 PMC8872699 35223101

[49] Somasundaram S , Edmund NA , Moore DT , Small GW , Shi YY , Orlowski RZ . Dietary curcumin inhibits chemotherapy‐induced apoptosis in models of human breast cancer. Cancer Res. 2002;62:3868‐3875. 12097302

[50] Morrissey C , Watson RWG . Phytoestrogens and prostate cancer. Curr Drug Targets. 2005;4:231‐241. 10.2174/1389450033491154 12643473

[51] Torrens‐Mas M , Roca P . Phytoestrogens for cancer prevention and treatment. Biology. 2020;9:1‐19. 10.3390/biology9120427 PMC7759898 33261116

[52] Sohel M , Sultana H , Sultana T , et al. Chemotherapeutics activities of dietary phytoestrogens against prostate cancer: from observational to clinical studies. Curr Pharm Des.2022;28:1561‐1580 35652403 10.2174/1381612828666220601153426

[53] Liu RH . Potential synergy of phytochemicals in cancer prevention: mechanism of action. J Nutr. 2004;134:3479S‐3485S. 15570057 10.1093/jn/134.12.3479S

[54] Ammar NS , Elhaes H , Ibrahim HS , El Hotaby W , Ibrahim MA . A novel structure for removal of pollutants from wastewater. Spectrochim Acta A Mol Biomol Spectrosc. 2014;121:216‐223. doi:10.1016/j.saa.2013.10.063 24239765

[55] Jiang D , Rasul A , Batool R , et al. Potential anticancer properties and mechanisms of action of Formononetin. Biomed Res Int. 2019;2019:1‐11. 10.1155/2019/5854315 PMC6699357 31467899

[56] Tuli HS , Tuorkey MJ , Thakral F , et al. Molecular mechanisms of action of genistein in cancer: recent advances. Front Pharmacol. 2019;10:1‐16. 31866857 10.3389/fphar.2019.01336 PMC6910185

[57] Burns J , Yokota T , Ashihara H , Lean MEJ , Crozier A . Plant foods and herbal sources of resveratrol. J Agric Food Chem. 2002;50:3337‐3340. 12010007 10.1021/jf0112973

[58] Abdel‐Aal ESM , Akhtar H , Zaheer K , Ali R . Dietary sources of lutein and zeaxanthin carotenoids and their role in eye health. Nutrients. 2013;5:1169‐1185. 23571649 10.3390/nu5041169 PMC3705341

[59] Amare DE . Anti‐cancer and other biological effects of a dietary compound 3,3′‐diindolylmethane supplementation: a systematic review of human clinical trials. Nutr Diet Suppl. 2020;12:123‐137.

[60] Dabeek WM , Marra MV . Dietary quercetin and kaempferol: bioavailability and potential cardiovascular‐related bioactivity in humans. Nutrients. 2019;11:2088. 31557798 10.3390/nu11102288 PMC6835347

[61] Orlikova B , Tasdemir D , Golais F , Dicato M , Diederich M . Dietary chalcones with chemopreventive and chemotherapeutic potential. Genes Nutr. 2011;6:125‐147. 21484163 10.1007/s12263-011-0210-5 PMC3092904

[62] Mourvaki E , Stefania G , Rossi R , Rufini S . Passionflower fruit‐a “new” source of lycopene? J Med Food. 2005;8:104‐106. 15857218 10.1089/jmf.2005.8.104

[63] Salehi B , Fokou P , Sharifi‐Rad M , et al. The therapeutic potential of naringenin: a review of clinical trials. Pharmaceuticals. 2019;12:11. 30634637 10.3390/ph12010011 PMC6469163

[64] Gupta SC , Prasad S , Sethumadhavan DR , Nair MS , Mo YY , Aggarwal BB . Nimbolide, a limonoid triterpene, inhibits growth of human colorectal cancer xenografts by suppressing the proinflammatory microenvironment. Clin Cancer Res. 2013;19:4465‐4476. 23766363 10.1158/1078-0432.CCR-13-0080 PMC4220790

[65] Basu P , Kumar GS . Sanguinarine and its role in chronic diseases. Adv Exp Med Biol. 2016;928:155‐172. 27671816 10.1007/978-3-319-41334-1_7

[66] Zhang KJ , Gu QL , Yang K , Ming XJ , Wang JX . Anticarcinogenic effects of α ‐mangostin: a review. Planta Med. 2017;83:188‐202. 27824406 10.1055/s-0042-119651

[67] Sun Y , Tan YJ , Lu ZZ , et al. Arctigenin inhibits liver cancer tumorigenesis by inhibiting gankyrin expression via C/EBPα and PPARα. Front Pharmacol. 2018;9:1‐12. 29636686 10.3389/fphar.2018.00268 PMC5880935

[68] Gao J , Liu ZJ , Chen T , Zhao D . Pharmaceutical properties of calycosin, the major bioactive isoflavonoid in the dry root extract of *Radix astragali* . Pharm Biol. 2014;52:1217‐1222. 24635389 10.3109/13880209.2013.879188

[69] Cao K , Xu J , Pu W , et al. Punicalagin, an active component in pomegranate, ameliorates cardiac mitochondrial impairment in obese rats via AMPK activation. Sci Rep. 2015;5:1‐12. 10.1038/srep14014 PMC4642696 26369619

[70] Peñalvo JL , Heinonen SM , Aura AM , Adlercreutz H . Dietary sesamin is converted to enterolactone in humans. J Nutr. 2005;135:1056‐1062. 15867281 10.1093/jn/135.5.1056

[71] Abu‐Lafi S , Makhamra S , Rayan I , et al. Sesamin from *Cuscuta palaestina* natural plant extracts: directions for new prospective applications. PLoS ONE. 2018;13:e0195707. 29634770 10.1371/journal.pone.0195707 PMC5892908

[72] Pashaei‐Asl F , Pashaei‐Asl R , Khodadadi K , Akbarzadeh A , Ebrahimie E , Pashaiasl M . Enhancement of anticancer activity by silibinin and paclitaxel combination on the ovarian cancer. Artif Cells Nanomed Biotechnol. 2018;46:1483‐1487. 28884602 10.1080/21691401.2017.1374281

[73] Harn HJ , Chuang HM , Chang LF , et al. Taiwanin a targets non‐steroidal anti‐inflammatory drug‐activated gene‐1 in human lung carcinoma. Fitoterapia. 2014;99:227‐235. 25173462 10.1016/j.fitote.2014.08.020

[74] Tai MC , Tsang SY , Chang LYF , Xue H . Therapeutic potential of wogonin: a naturally occurring flavonoid. CNS Drug Rev. 2005;11:141‐150. 16007236 10.1111/j.1527-3458.2005.tb00266.x PMC6741757

[75] Fujiki H , Yoshizawa S , Horiuchi T , et al. Anticarcinogenic effects of (−)‐epigallocatechin gallate. Prev Med. 1992;21:503‐509. 1409491 10.1016/0091-7435(92)90057-o

[76] Jin P , Li M , Xu G , et al. Role of (−)‐epigallocatechin‐3‐gallate in the osteogenic differentiation of human bone marrow mesenchymal stem cells: an enhancer or an inducer? Exp Ther Med. 2015;10:828‐834. 26622401 10.3892/etm.2015.2579 PMC4508993

[77] Shankar E , Goel A , Gupta K , Gupta S . Plant flavone apigenin: an emerging anticancer agent. Curr Pharmacol Rep. 2017;3:423‐446. 29399439 10.1007/s40495-017-0113-2 PMC5791748

[78] Liu J , Lee J , Hernandez MAS , Mazitschek R , Ozcan U . Treatment of obesity with celastrol. Cell. 2015;161:999‐1011. 26000480 10.1016/j.cell.2015.05.011 PMC4768733

[79] Bhagwat S , Haytowitz DB , Holden JM . USDA database for the isoflavone content of selected foods, release 2.0. Vol 15. U.S. Department of Agriculture; 2008.

[80] Hewlings SJ , Kalman DS . Curcumin: a review of its effects on human health. Foods. 2017;6:92. doi:10.3390/foods6100092 29065496 PMC5664031

[81] Aires V , Limagne E , Cotte AK , Latruffe N , Ghiringhelli F , Delmas D . Resveratrol metabolites inhibit human metastatic colon cancer cells progression and synergize with chemotherapeutic drugs to induce cell death. Mol Nutr Food Res. 2013;57:1170‐1181. 23495229 10.1002/mnfr.201200766

[82] Dong X , Fu J , Yin X . Emodin: a review of its pharmacology, toxicity and pharmacokinetics. Phytother Res. 2016;30:1207‐1218. 27188216 10.1002/ptr.5631 PMC7168079

[83] Moradzadeh M , Hosseini A , Erfanian S , Rezaei H . Epigallocatechin‐3‐gallate promotes apoptosis in human breast cancer T47D cells through down‐regulation of PI3K/AKT and telomerase. Pharmacol Rep. 2017;69:924‐928. 28646740 10.1016/j.pharep.2017.04.008

[84] Sedlacek HH , Czech J , Naik R , et al. Flavopiridol (L86 8275; NSC 649890), a new kinase inhibitor for tumor therapy. Int J Oncol. 1996;9:1143‐1168. doi:10.3892/ijo.9.6.1143 21541623

[85] Lyu X , Xu X , Song A , Guo J , Zhang Y , Zhang Y . Ginsenoside Rh1 inhibits colorectal cancer cell migration and invasion in vitro and tumor growth in vivo. Oncol Lett. 2019;18:4160‐4166. 31579419 10.3892/ol.2019.10742 PMC6757309

[86] Christensen LP . Chapter 1 Ginsenosides. Chemistry, biosynthesis, analysis, and potential health effects. Adv Food Nutr Res. 2008;55:1‐99. 10.1016/S1043-4526(08)00401-4 18772102

[87] Kong LD , Zhang Y , Pan X , Tan RX , Cheng CHK . Inhibition of xanthine oxidase by liquiritigenin and isoliquiritigenin isolated from *Sinofranchetia chinensis* . Cell Mol Life Sci. 2000;57:500‐505. 10823249 10.1007/PL00000710 PMC11146775

[88] Park YJ , Choi CI , Chung KH , Kim KH . Pharbilignan C induces apoptosis through a mitochondria‐mediated intrinsic pathway in human breast cancer cells. Bioorg Med Chem Lett. 2016;26:4645‐4649. 27575473 10.1016/j.bmcl.2016.08.054

[89] McCormack D , McFadden D . Pterostilbene and cancer: current review. J Surg Res. 2012;173:e53‐e61. 22099605 10.1016/j.jss.2011.09.054

[90] Anand David AV , Arulmoli R , Parasuraman S . Overviews of biological importance of quercetin: a bioactive flavonoid. Pharmacogn Rev. 2016;10:84‐89. 28082789 10.4103/0973-7847.194044 PMC5214562

[91] Popovich DG , Kitts DD . Generation of ginsenosides Rg3 and Rh2 from North American ginseng. Phytochemistry. 2004;65:337‐344. 14751305 10.1016/j.phytochem.2003.11.020

[92] Petersen M , Simmonds MSJ . Rosmarinic acid. Phytochemistry. 2003;62:121‐125. 12482446 10.1016/s0031-9422(02)00513-7

[93] Fujita Y . Shikonin: production by plant (*Lithospermum erythrorhizon*) cell cultures. In Bajaj Y.P.S. eds. Medicinal and Aromatic Plants I. Springer; 1988:225‐236.

[94] Herr I , Lozanovski V , Houben P , Schemmer P , Büchler MW . Sulforaphane and related mustard oils in focus of cancer prevention and therapy. Wien Med Wochenschr. 2013;163:80‐88. 23224634 10.1007/s10354-012-0163-3

[95] Goyal SN , Prajapati CP , Gore PR , et al. Therapeutic potential and pharmaceutical development of thymoquinone: a multitargeted molecule of natural origin. Front Pharmacol. 2017;8:1‐19. 28983249 10.3389/fphar.2017.00656 PMC5613109

[96] Guzman JR , Koo JS , Goldsmith JR , Mühlbauer M , Narula A , Jobin C . Oxymatrine prevents NF‐κB nuclear translocation and ameliorates acute intestinal inflammation. Sci Rep. 2013;3:1629. 23568217 10.1038/srep01629 PMC3620667

[97] Flescher E . Jasmonates in cancer therapy. Cancer Lett. 2007;245:1‐10. 16600475 10.1016/j.canlet.2006.03.001

[98] Grynkiewicz G , Demchuk OM . New perspectives for fisetin. Front Chem. 2019;7:1‐10. 31750288 10.3389/fchem.2019.00697 PMC6842927

[99] Majeed W , Aslam B , Javed I , et al. Breast cancer: major risk factors and recent developments in treatment. Asian Pac J Cancer Prev. 2014;15:3353‐3358. 24870721 10.7314/apjcp.2014.15.8.3353

[100] Catalano E . Role of phytochemicals in the chemoprevention of tumors. arXiv Prepr. arXiv1605.04519 2016.

[101] Wu XY , Xu H , Wu ZF , et al. Formononetin, a novel FGFR2 inhibitor, potently inhibits angiogenesis and tumor growth in preclinical models. Oncotarget. 2015;6:44563‐44578. 26575424 10.18632/oncotarget.6310 PMC4792576

[102] Yokota T , Matsuzaki Y , Koyama M , et al. Sesamin, a lignan of sesame, down‐regulates cyclin D1 protein expression in human tumor cells. Cancer Sci. 2007;98:1447‐1453. 17640297 10.1111/j.1349-7006.2007.00560.x PMC11159746

[103] Liu Q , Loo WTY , Sze SCW , Tong Y . Curcumin inhibits cell proliferation of MDA‐MB‐231 and BT‐483 breast cancer cells mediated by down‐regulation of NFκB, cyclinD and MMP‐1 transcription. Phytomedicine. 2009;16:916‐922. 19524420 10.1016/j.phymed.2009.04.008

[104] Chen J , Duan Y , Zhang X , Ye Y , Ge B , Chen J . Genistein induces apoptosis by the inactivation of the IGF‐1R/p‐Akt signaling pathway in MCF‐7 human breast cancer cells. Food Funct. 2015;6:995‐1000. 25675448 10.1039/c4fo01141d

[105] Takeshima M , Ono M , Higuchi T , Chen C , Hara T , Nakano S . Anti‐proliferative and apoptosis‐inducing activity of lycopene against three subtypes of human breast cancer cell lines. Cancer Sci. 2014;105:252‐257. 24397737 10.1111/cas.12349 PMC4317951

[106] Scheckel KA , Degner SC , Romagnolo DF . Rosmarinic acid antagonizes activator protein‐1‐dependent activation of cyclooxygenase‐2 expression in human cancer and nonmalignant cell lines. J Nutr. 2008;138:2098‐2105. 18936204 10.3945/jn.108.090431 PMC3151436

[107] Pirouzpanah MB , Sabzichi M , Pirouzpanah S , Chavoshi H , Samadi N . Silibilin‐induces apoptosis in breast cancer cells by modulating p53, p21, bak and bcl‐xl pathways. Asian Pac J Cancer Prev. 2015;16:2087‐2092. 25773855 10.7314/apjcp.2015.16.5.2087

[108] Harrison ME , Power Coombs MR , Delaney LM , Hoskin DW . Exposure of breast cancer cells to a subcytotoxic dose of apigenin causes growth inhibition, oxidative stress, and hypophosphorylation of Akt. Exp Mol Pathol. 2014;97:211‐217. 25019465 10.1016/j.yexmp.2014.07.006

[109] Mali AV , Joshi AA , Hegde MV , Kadam SS . Enterolactone suppresses proliferation, migration and metastasis of MDA‐MB‐231 breast cancer cells through inhibition of uPA induced plasmin activation and MMPs‐mediated ECM remodeling. Asian Pac J Cancer Prev. 2017;18:905‐915. 28545187 10.22034/APJCP.2017.18.4.905 PMC5494239

[110] Huynh DTN , Jin Y , Myung CS , Heo KS . Ginsenoside rh1 induces mcf‐7 cell apoptosis and autophagic cell death through ros‐mediated akt signaling. Cancers. 2021;13:1892. 33920802 10.3390/cancers13081892 PMC8071122

[111] Jin S , Zhang QY , Kang XM , Wang JX , Zhao WH . Daidzein induces MCF‐7 breast cancer cell apoptosis via the mitochondrial pathway. Ann Oncol. 2010;21:263‐268. 19889614 10.1093/annonc/mdp499

[112] Pooladanda V , Bandi S , Mondi SR , Gottumukkala KM , Godugu C . Nimbolide epigenetically regulates autophagy and apoptosis in breast cancer. Toxicol In Vitro. 2018;51:114‐128. 29778718 10.1016/j.tiv.2018.05.010

[113] Peng SJ , Li J , Zhou Y , et al. In vitro effects and mechanisms of lycopene in MCF‐7 human breast cancer cells. Genet Mol Res. 2017;16:13. 10.4238/gmr16029434 28407181

[114] Choi WY , Kim GY , Lee WH , Choi YH . Sanguinarine, a benzophenanthridine alkaloid, induces apoptosis in MDA‐MB‐231 human breast carcinoma cells through a reactive oxygen species‐mediated mitochondrial pathway. Chemotherapy. 2008;54:279‐287. 18667818 10.1159/000149719

[115] Chew BP , Brown CM , Park JS , Mixter PF . Dietary lutein inhibits mouse mammary tumor growth by regulating angiogenesis and apoptosis. Anticancer Res. 2003;23:3333‐3339. 12926072

[116] Yi X , Zuo J , Tan C , et al. Kaempferol, a flavonoid compound from gynura medica induced apoptosis and growth inhibition in MCF‐7 breast cancer cell. Afr J Tradit Complement Altern Med. 2016;13:210‐215. 28852738 10.21010/ajtcam.v13i4.27 PMC5566146

[117] Zu C , Zhang M , Xue H , et al. Emodin induces apoptosis of human breast cancer cells by modulating the expression of apoptosis‐related genes. Oncol Lett. 2015;10:2919‐2924. 26722264 10.3892/ol.2015.3646 PMC4665964

[118] Hahm ER , Moura MB , Kelley EE , Van Houten B , Shiva S , Singh SV . Withaferin A‐induced apoptosis in human breast cancer cells is mediated by reactive oxygen species. PLoS ONE. 2011;6:e23354. 21853114 10.1371/journal.pone.0023354 PMC3154436

[119] Mi C , Shi H , Ma J , et al. Celastrol induces the apoptosis of breast cancer cells and inhibits their invasion via downregulation of MMP‐9. Oncol Rep. 2014;32:2527‐2532. 25310109 10.3892/or.2014.3535

[120] Choi JA , Kim JY , Lee JY , et al. Induction of cell cycle arrest and apoptosis in human breast cancer cells by quercetin. Int J Oncol. 2001;19:837‐844. 11562764 10.3892/ijo.19.4.837

[121] Shyur LF , Lee SH , Chang ST , Lo CP , Kuo YH , Wang SY . Taiwanin a inhibits MCF‐7 cancer cell activity through induction of oxidative stress, upregulation of DNA damage checkpoint kinases, and activation of p53 and FasL/Fas signaling pathways. Phytomedicine. 2010;18:16‐24. 20637573 10.1016/j.phymed.2010.06.005

[122] Zafar A , Singh S , Naseem I . Cytotoxic activity of soy phytoestrogen coumestrol against human breast cancer MCF‐7 cells: insights into the molecular mechanism. Food Chem Toxicol. 2017;99:149‐161. 27913286 10.1016/j.fct.2016.11.034

[123] Kim SJ , Kim AK . Anti‐breast cancer activity of fine black ginseng (*Panax ginseng* Meyer) and ginsenoside Rg5. J Ginseng Res. 2015;39:125‐134. 26045685 10.1016/j.jgr.2014.09.003 PMC4452536

[124] Kim SH , Hwang KA , Choi KC . Treatment with kaempferol suppresses breast cancer cell growth caused by estrogen and triclosan in cellular and xenograft breast cancer models. J Nutr Biochem. 2016;28:70‐82. 26878784 10.1016/j.jnutbio.2015.09.027

[125] Rajput S , Kumar BNP , Dey KK , Pal I , Parekh A , Mandal M . Molecular targeting of Akt by thymoquinone promotes G1 arrest through translation inhibition of cyclin D1 and induces apoptosis in breast cancer cells. Life Sci. 2013;93:783‐790. 24044882 10.1016/j.lfs.2013.09.009

[126] Abaza MSI , Orabi KY , Al‐Quattan E , Al‐Attiyah RJ . Growth inhibitory and chemo‐sensitization effects of naringenin, a natural flavanone purified from *Thymus vulgaris*, on human breast and colorectal cancer. Cancer Cell Int. 2015;15:46. 26074733 10.1186/s12935-015-0194-0 PMC4464250

[127] Jang SY , Lee JK , Jang EH , Jeong SY , Kim JH . Shikonin blocks migration and invasion of human breast cancer cells through inhibition of matrix metalloproteinase‐9 activation. Oncol Rep. 2014;31:2827‐2833. 24789371 10.3892/or.2014.3159

[128] Li Y , Bhuiyan M , Alhasan S , Senderowicz AM , Sarkar FH . Induction of apoptosis and inhibition of c‐erbB‐2 in breast cancer cells by flavopiridol. Clin Cancer Res. 2000;6:223‐229. 10656453

[129] Jiang C , Agarwal R , Lü J . Anti‐angiogenic potential of a cancer chemopreventive flavonoid antioxidant, silymarin: inhibition of key attributes of vascular endothelial cells and angiogenic cytokine secretion by cancer epithelial cells. Biochem Biophys Res Commun. 2000;276:371‐378. 11006131 10.1006/bbrc.2000.3474

[130] Zong H , Wang F , Fan QX , Wang LX . Curcumin inhibits metastatic progression of breast cancer cell through suppression of urokinase‐type plasminogen activator by NF‐kappa B signaling pathways. Mol Biol Rep. 2012;39:4803‐4808. 21947854 10.1007/s11033-011-1273-5

[131] Maxwell T , Chun SY , Lee KS , Kim S , Nam KS . The anti‐metastatic effects of the phytoestrogen arctigenin on human breast cancer cell lines regardless of the status of ER expression. Int J Oncol. 2017;50:727‐735. 28035371 10.3892/ijo.2016.3825

[132] Kim SY , Lee IS , Moon A . 2‐Hydroxychalcone and xanthohumol inhibit invasion of triple negative breast cancer cells. Chem Biol Interact. 2013;203:565‐572. 23562496 10.1016/j.cbi.2013.03.012

[133] Zhao X , Wang Q , Yang S , et al. Quercetin inhibits angiogenesis by targeting calcineurin in the xenograft model of human breast cancer. Eur J Pharmacol. 2016;781:60‐68. 27041643 10.1016/j.ejphar.2016.03.063

[134] Zhang Y , Liu QZ , Xing SP , Zhang JL . Inhibiting effect of Endostar combined with ginsenoside Rg3 on breast cancer tumor growth in tumor‐bearing mice. Asian Pac J Trop Med. 2016;9:180‐183. 26919952 10.1016/j.apjtm.2016.01.010

[135] Bao C , Ko J , Park HC , et al. Sulforaphane inhibited tumor necrosis factor‐α induced migration and invasion in estrogen receptor negative human breast cancer cells. Food Sci Biotechnol. 2015;24:347‐351.

[136] Kil WH , Kim SM , Lee JE , Park KS , Nam SJ . Anticancer effect of silibinin on the xenograft model using MDA‐MB‐468 breast cancer cells. Ann Surg Treat Res. 2014;87:167‐173. 25317410 10.4174/astr.2014.87.4.167 PMC4196436

[137] Wang KL , Hsia SM , Chan CJ , et al. Inhibitory effects of isoliquiritigenin on the migration and invasion of human breast cancer cells. Expert Opin Ther Targets. 2013;17:337‐349. 23327692 10.1517/14728222.2013.756869

[138] Rajput S , Kumar BNP , Banik P , Parida S , Mandal M . Thymoquinone restores radiation‐induced TGF‐β expression and abrogates EMT in chemoradiotherapy of breast cancer cells. J Cell Physiol. 2015;230:620‐629. 25164250 10.1002/jcp.24780

[139] Toi M , Bando H , Ramachandran C , et al. Preliminary studies on the anti‐angiogenic potential of pomegranate fractions in vitro and in vivo. Angiogenesis. 2003;6:121‐128. 14739618 10.1023/B:AGEN.0000011802.81320.e4

[140] Gu JW , Makey KL , Tucker KB , et al. EGCG, a major green tea catechin suppresses breast tumor angiogenesis and growth via inhibiting the activation of HIF‐1α and NFκB, and VEGF expression. Vasc Cell. 2013;5:1‐10. 23638734 10.1186/2045-824X-5-9 PMC3649947

[141] Riby JE , Firestone GL , Bjeldanes LF . 3,3′‐Diindolylmethane reduces levels of HIF‐1α and HIF‐1 activity in hypoxic cultured human cancer cells. Biochem Pharmacol. 2008;75:1858‐1867. 18329003 10.1016/j.bcp.2008.01.017 PMC2387239

[142] Song X , Yao J , Wang F , et al. Wogonin inhibits tumor angiogenesis via degradation of HIF‐1α protein. Toxicol Appl Pharmacol. 2013;271:144‐155. 23707765 10.1016/j.taap.2013.04.031

[143] Wu CH , Hong BH , Ho CT , Yen GC . Targeting cancer stem cells in breast cancer: potential anticancer properties of 6‐shogaol and pterostilbene. J Agric Food Chem. 2015;63:2432‐2441. 25686711 10.1021/acs.jafc.5b00002

[144] Burnett JP , Lim G , Li Y , et al. Sulforaphane enhances the anticancer activity of taxanes against triple negative breast cancer by killing cancer stem cells. Cancer Lett. 2017;394:52‐64. 28254410 10.1016/j.canlet.2017.02.023 PMC8892390

[145] Kim SH , Sehrawat A , Singh SV . Dietary chemopreventative benzyl isothiocyanate inhibits breast cancer stem cells in vitro and in vivo. Cancer Prev Res. 2013;6:782‐790. 10.1158/1940-6207.CAPR-13-0100 PMC3737245 23661606

[146] Fu Y , Chang H , Peng X , et al. Resveratrol inhibits breast cancer stem‐like cells and induces autophagy via suppressing Wnt/β‐catenin signaling pathway. PLoS ONE. 2014;9:e102535. 25068516 10.1371/journal.pone.0102535 PMC4113212

[147] Colacino JA , McDermott SP , Sartor MA , Wicha MS , Rozek LS . Transcriptomic profiling of curcumin‐treated human breast stem cells identifies a role for stearoyl‐coa desaturase in breast cancer prevention. Breast Cancer Res Treat. 2016;158:29‐41. 27306423 10.1007/s10549-016-3854-4 PMC5831404

[148] Pan X , Zhao B , Song Z , Han S , Wang M . Estrogen receptor‐α36 is involved in epigallocatechin‐3‐gallate induced growth inhibition of ER‐negative breast cancer stem/progenitor cells. J Pharmacol Sci. 2016;130:85‐93. 26810571 10.1016/j.jphs.2015.12.003

[149] Aharoni S , Lati Y , Aviram M , Fuhrman B . Pomegranate juice polyphenols induce a phenotypic switch in macrophage polarization favoring a M2 anti‐inflammatory state. Biofactors. 2015;41:44‐51. 25650983 10.1002/biof.1199

[150] Yoon H , Rui HL . Effect of selected phytochemicals and apple extracts on NF‐κB activation in human breast cancer MCF‐7 cells. J Agric Food Chem. 2007;55:3167‐3173. 17373813 10.1021/jf0632379

[151] Banerjee S , Bueso‐Ramos C , Aggarwal BB . Suppression of 7,12‐dimethylbenz(a)anthracene‐induced mammary carcinogenesis in rats by resveratrol: role of nuclear factor‐κB, cyclooxygenase 2, and matrix metalloprotease 9. Cancer Res. 2002;62:4945‐4954. 12208745

[152] Subbaramaiah K , Sue E , Bhardwaj P , et al. Dietary polyphenols suppress elevated levels of proin flammatory mediators and aromatase in the mammary gland of obese mice. Cancer Prev Res. 2013;6:886‐897. 10.1158/1940-6207.CAPR-13-0140 PMC3767430 23880231

[153] Kumar U , Sharma U , Rathi G . Reversal of hypermethylation and reactivation of glutathione S‐transferase pi 1 gene by curcumin in breast cancer cell line. Tumour Biol. 2017;39:1010428317692258. 28222671 10.1177/1010428317692258

[154] Wang Y , Lee KW , Chan FL , Chen S , Leung LK . The red wine polyphenol resveratrol displays bilevel inhibition on aromatase in breast cancer cells. Toxicol Sci. 2006;92:71‐77. 16611627 10.1093/toxsci/kfj190

[155] Kim HN , Kim DH , Kim EH , et al. Sulforaphane inhibits phorbol ester‐stimulated IKK‐NF‐κB signaling and COX‐2 expression in human mammary epithelial cells by targeting NF‐κB activating kinase and ERK. Cancer Lett. 2014;351:41‐49. 24747121 10.1016/j.canlet.2014.03.037

[156] Kim S , Kim SH , Hur SM , et al. Silibinin prevents TPA‐induced MMP‐9 expression by down‐regulation of COX‐2 in human breast cancer cells. J Ethnopharmacol. 2009;126:252‐257. 19715751 10.1016/j.jep.2009.08.032

[157] Zheng H , Li Y , Wang Y , et al. Downregulation of COX‐2 and CYP 4A signaling by isoliquiritigenin inhibits human breast cancer metastasis through preventing anoikis resistance, migration and invasion. Toxicol Appl Pharmacol. 2014;280:10‐20. 25094029 10.1016/j.taap.2014.07.018

[158] Moreira L , Araújo I , Costa T , et al. Quercetin and epigallocatechin gallate inhibit glucose uptake and metabolism by breast cancer cells by an estrogen receptor‐independent mechanism. Exp Cell Res. 2013;319:1784‐1795. 23664836 10.1016/j.yexcr.2013.05.001

[159] Seo HS , Choi HS , Choi HS , et al. Phytoestrogens induce apoptosis via extrinsic pathway, inhibiting nuclear factor‐κB signaling in HER2‐overexpressing breast cancer cells. Anticancer Res. 2011;31:3301‐3313. 21965740

[160] Chen J , Zeng J , Xin M , Huang W , Chen X . Formononetin induces cell cycle arrest of human breast cancer cells via IGF1/PI3K/Akt pathways in vitro and in vivo. Horm Metab Res. 2011;43:681‐686. 21932171 10.1055/s-0031-1286306

[161] Chen J , Hou R , Zhang X , Ye Y , Wang Y , Tian J . Calycosin suppresses breast cancer cell growth via ERβ‐dependent regulation of IGF‐1R, p38 MAPK and PI3K/Akt pathways. PLoS ONE. 2014;9:e91245. 24618835 10.1371/journal.pone.0091245 PMC3949755

[162] Scherbakov AM , Andreeva OE . Apigenin inhibits growth of breast cancer cells: the role of ERα and HER2/neu. Acta Nat. 2015;7:133‐139. PMC4610175 26483970

[163] Kim TH , Woo JS , Kim YK , Kim KH . Silibinin induces cell death through reactive oxygen species‐dependent downregulation of Notch‐1/ERK/Akt signaling in human breast cancer cells. J Pharmacol Exp Ther. 2014;349:268‐278. 24472723 10.1124/jpet.113.207563

[164] Wakimoto R , Ono M , Takeshima M , Higuchi T , Nakano S . Differential anticancer activity of pterostilbene against three subtypes of human breast cancer cells. Anticancer Res. 2017;37:6153‐6159. 29061796 10.21873/anticanres.12064

[165] Hatkevich T , Ramos J , Santos‐Sanchez I , Patel YM . A naringenin‐tamoxifen combination impairs cell proliferation and survival of MCF‐7 breast cancer cells. Exp Cell Res. 2014;327:331‐339. 24881818 10.1016/j.yexcr.2014.05.017

[166] Kritsanawong S , Innajak S , Imoto M , Watanapokasin R . Antiproliferative and apoptosis induction of α‐mangostin in T47D breast cancer cells. Int J Oncol. 2016;48:2155‐2165. 26892433 10.3892/ijo.2016.3399

[167] Li Y , Chen H , Hardy TM , Tollefsbol TO . Epigenetic regulation of multiple tumor‐related genes leads to suppression of breast tumorigenesis by dietary genistein. PLoS ONE. 2013;8:e54369. 23342141 10.1371/journal.pone.0054369 PMC3544723

[168] King‐Batoon A , Leszczynska JM , Klein CB . Modulation of gene methylation by genistein or lycopene in breast cancer cells. Environ Mol Mutagen. 2008;49:36‐45. 18181168 10.1002/em.20363

[169] Liu Y , Zhou J , Hu Y , Wang J , Yuan C . Curcumin inhibits growth of human breast cancer cells through demethylation of DLC1 promoter. Mol Cell Biochem. 2017;425:47‐58. 27830358 10.1007/s11010-016-2861-4

[170] Mirza S , Sharma G , Parshad R , Gupta SD , Pandya P , Ralhan R . Expression of DNA methyltransferases in breast cancer patients and to analyze the effect of natural compounds on DNA methyltransferases and associated proteins. J Breast Cancer. 2013;16:23‐31. 23593078 10.4048/jbc.2013.16.1.23 PMC3625766

[171] Meeran SM , Patel SN , Li Y , Shukla S , Tollefsbol TO . Bioactive dietary supplements reactivate ER expression in ER‐negative breast cancer cells by active chromatin modifications. PLoS ONE. 2012;7:e37748. 22662208 10.1371/journal.pone.0037748 PMC3360625

[172] Delazar A , Asnaashari S , Nikkhah E , Asgharian P . Phytochemical analysis and antiproliferative activity of the aerial parts of Scrophularia subaphylla. Res Pharm Sci. 2019;14:263‐272. 31160904 10.4103/1735-5362.258495 PMC6540926

[173] Parrish AB , Freel CD , Kornbluth S . Cellular mechanisms controlling caspase activation and function. Cold Spring Harb Perspect Biol. 2013;5:1‐24. 10.1101/cshperspect.a008672 PMC3660825 23732469

[174] Safarzadeh E , Shotorbani SS , Baradaran B . Herbal medicine as inducers of apoptosis in cancer treatment. Adv Pharm Bull. 2014;4:421‐427. 25364657 10.5681/apb.2014.062 PMC4213780

[175] Papaliagkas V , Anogianaki A , Anogianakis G , Ilonidis G . The proteins and the mechanisms of apoptosis: a mini‐review of the fundamentals. Hippokratia. 2007;11:108‐113. 19582203 PMC2658792

[176] Elmore S . Apoptosis: a review of programmed cell death. Toxicol Pathol. 2007;35:495‐516. 17562483 10.1080/01926230701320337 PMC2117903

[177] Hongmei Z . Extrinsic and intrinsic apoptosis signal pathway review. Apoptosis and Medicine. Molecular Genetics; 2012. doi:10.5772/50129

[178] Lv ZD , Liu XP , Zhao WJ , et al. Curcumin induces apoptosis in breast cancer cells and inhibits tumor growth in vitro and in vivo. Int J Clin Exp Pathol. 2014;7:2818‐2824. 25031701 PMC4097278

[179] Venkatadri R , Muni T , Iyer AKV , Yakisich JS , Azad N . Role of apoptosis‐related miRNAs in resveratrol‐induced breast cancer cell death. Cell Death Dis. 2016;7:e2104. 26890143 10.1038/cddis.2016.6 PMC5399194

[180] Şakalar Ç , İzgi K , İskender B , et al. The combination of thymoquinone and paclitaxel shows anti‐tumor activity through the interplay with apoptosis network in triple‐negative breast cancer. Tumor Biol. 2016;37:4467‐4477. 10.1007/s13277-015-4307-0 26500095

[181] Seo HS , Ku JM , Choi HS , et al. Induction of caspase‐dependent apoptosis by apigenin by inhibiting STAT3 signaling in HER2‐overexpressing MDA‐MB‐453 breast cancer cells. Anticancer Res. 2014;34:2869‐2882. 24922650

[182] Kastan MB , Bartek J . Cell‐cycle checkpoints and cancer. Nature. 2004;432:316‐323. 15549093 10.1038/nature03097

[183] Barnum KJ , O'Connell MJ . Cell cycle regulation by checkpoints. Methods Mol Biol. 2014;1170:29‐40. 24906307 10.1007/978-1-4939-0888-2_2 PMC4990352

[184] Horak ER , Klenk N , Leek R , et al. Angiogenesis, assessed by platelet/endothelial cell adhesion molecule antibodies, as indicator of node metastases and survival in breast cancer. Lancet. 1992;340:1120‐1124. 1279332 10.1016/0140-6736(92)93150-l

[185] Way TD , Lin JK . Role of HER2/HER3 co‐receptor in breast carcinogenesis. Future Oncol. 2005;1:841‐849. 16556064 10.2217/14796694.1.6.841

[186] Papi A , Guarnieri T , Storci G , et al. Nuclear receptors agonists exert opposing effects on the inflammation dependent survival of breast cancer stem cells. Cell Death Differ. 2012;19:1208‐1219. 22261616 10.1038/cdd.2011.207 PMC3374082

[187] Muz B , de la Puente P , Azab F , Azab AK . The role of hypoxia in cancer progression, angiogenesis, metastasis, and resistance to therapy. Hypoxia. 2015;3:83‐92. 27774485 10.2147/HP.S93413 PMC5045092

[188] Wang W , Dai M , Zhu C , et al. Synthesis and biological activity of novel shikonin analogues. Bioorg Med Chem Lett. 2009;19:735‐737. 19111464 10.1016/j.bmcl.2008.12.032

[189] Ríos‐Arrabal S , Artacho‐Cordón F , León J , et al. Involvement of free radicals in breast cancer. Springerplus. 2013;2:1‐12. 24024092 10.1186/2193-1801-2-404 PMC3765596

[190] Bhattacharyya A , Chattopadhyay R , Mitra S , Crowe SE . Oxidative stress: an essential factor in the pathogenesis of gastrointestinal mucosal diseases. Physiol Rev. 2014;94:329‐354. 24692350 10.1152/physrev.00040.2012 PMC4044300

[191] Pham‐Huy LA , He H , Pham‐Huy C . Free radicals, antioxidants in disease and health. Int J Biomed Sci. 2008;4:89‐96. 23675073 PMC3614697

[192] Singh B , Shoulson R , Chatterjee A , et al. Resveratrol inhibits estrogen‐induced breast carcinogenesis through induction of NRF2‐mediated protective pathways. Carcinogenesis. 2014;35:1872‐1880. 24894866 10.1093/carcin/bgu120 PMC4123650

[193] Mishra P , Kale RK , Kar A . Chemoprevention of mammary tumorigenesis and chemomodulation of the antioxidative enzymes and peroxidative damage in prepubertal Sprague Dawley rats by Biochanin A. Mol Cell Biochem. 2008;312:1‐9. 18273562 10.1007/s11010-008-9714-8

[194] Nadal‐Serrano M , Pons DG , Sastre‐Serra J , Blanquer‐Rosselló MM , Roca P , Oliver J . Genistein modulates oxidative stress in breast cancer cell lines according to ERa/ERβ ratio: effects on mitochondrial functionality, sirtuins, uncoupling protein 2 and antioxidant enzymes. Int J Biochem Cell Biol. 2013;45:2045‐2051. 23871935 10.1016/j.biocel.2013.07.002

[195] Fan S , Meng Q , Saha T , Sarkar FH , Rosen EM . Low concentrations of diindolylmethane, a metabolite of indole‐3‐carbinol, protect against oxidative stress in a BRCA1‐dependent manner. Cancer Res. 2009;69:6083‐6091. 19622773 10.1158/0008-5472.CAN-08-3309 PMC2777684

[196] Manuel Iglesias J , Beloqui I , Garcia‐Garcia F , et al. Mammosphere formation in breast carcinoma cell lines depends upon expression of E‐cadherin. PLoS ONE. 2013;8:e77281. 24124614 10.1371/journal.pone.0077281 PMC3790762

[197] Dandawate PR , Subramaniam D , Jensen RA , Anant S . Targeting cancer stem cells and signaling pathways by phytochemicals: novel approach for breast cancer therapy. Seminars in Cancer Biology. Elsevier; 2016; Vol 40‐41:192‐208. 10.1016/j.semcancer.2016.09.001 PMC5565737 27609747

[198] Li Y , Zhang T , Korkaya H , et al. Sulforaphane, a dietary component of broccoli/broccoli sprouts, inhibits breast cancer stem cells. Clin Cancer Res. 2010;16:2580‐2590. 20388854 10.1158/1078-0432.CCR-09-2937 PMC2862133

[199] Kakarala M , Brenner DE , Korkaya H , et al. Targeting breast stem cells with the cancer preventive compounds curcumin and piperine. Breast Cancer Res Treat. 2010;122:777‐785. 19898931 10.1007/s10549-009-0612-x PMC3039120

[200] Chen L , Deng H , Cui H , et al. Inflammatory responses and inflammation‐associated diseases in organs. Oncotarget. 2018;9:7204‐7218. 29467962 10.18632/oncotarget.23208 PMC5805548

[201] Lin EY , Pollard JW . Role of infiltrated leucocytes in tumour growth and spread. Br J Cancer. 2004;90:2053‐2058. 15164120 10.1038/sj.bjc.6601705 PMC2410285

[202] Xu L , Yi HG , Wu Z , et al. Activation of mucosal mast cells promotes inflammation‐related colon cancer development through recruiting and modulating inflammatory CD11b+Gr1+ cells. Cancer Lett. 2015;364:173‐180. 25986744 10.1016/j.canlet.2015.05.014

[203] Issa AY , Volate SR , Wargovich MJ . The role of phytochemicals in inhibition of cancer and inflammation: new directions and perspectives. J Food Compos Anal. 2006;19:405‐419.

[204] Dharmappa KK , Mohamed R , Shivaprasad HV , Vishwanath BS . Genistein, a potent inhibitor of secretory phospholipase A2: a new insight in down regulation of inflammation. Inflammopharmacology. 2010;18:25‐31. 19894024 10.1007/s10787-009-0018-8

[205] Meybodi NM , Mortazavian AM , Monfared AB , Sohrabvandi S , Meybodi FA . Phytochemicals in cancer prevention: a review of the evidence. Iran J Cancer Prev. 2017;10:e7219.

[206] Chen B , Zhang Y , Wang Y , Rao J , Jiang X , Xu Z . Curcumin inhibits proliferation of breast cancer cells through Nrf2‐mediated down‐regulation of Fen1 expression. J Steroid Biochem Mol Biol. 2014;143:11‐18. 24486718 10.1016/j.jsbmb.2014.01.009

[207] Sinha D , Sarkar N , Biswas J , Bishayee A . Resveratrol for breast cancer prevention and therapy: preclinical evidence and molecular mechanisms. Semin Cancer Biol. 2016;40‐41:209‐232. 10.1016/j.semcancer.2015.11.001 26774195

[208] Licznerska BE , Szaefer H , Murias M , Bartoszek A , Baer‐Dubowska W . Modulation of CYP19 expression by cabbage juices and their active components: Indole‐3‐carbinol and 3,3′‐diindolylmethene in human breast epithelial cell lines. Eur J Nutr. 2013;52:1483‐1492. 23090135 10.1007/s00394-012-0455-9 PMC3715682

[209] Chamcheu JC et al. Role and therapeutic targeting of the PI3K/Akt/mTOR signaling pathway in skin cancer: a review of current status and future trends on natural and synthetic agents therapy. Cell. 2019;8:1‐13. 10.3390/cells8080803 PMC6721560 31370278

[210] Chestnut C , Subramaniam D , Dandawate P , et al. Targeting major signaling pathways of bladder cancer with phytochemicals: a review. Nutr Cancer. 2020;73:2249‐2271. doi:10.1080/01635581.2020.1856895 33305598

[211] Li Z , Li J , Mo B , et al. Genistein induces cell apoptosis in MDA‐MB‐231 breast cancer cells via the mitogen‐activated protein kinase pathway. Toxicol In Vitro. 2008;22:1749‐1753. 18761399 10.1016/j.tiv.2008.08.001

[212] Khan A , Aljarbou AN , Aldebasi YH , Faisal SM , Khan MA . Resveratrol suppresses the proliferation of breast cancer cells by inhibiting fatty acid synthase signaling pathway. Cancer Epidemiol. 2014;38:765‐772. 25448084 10.1016/j.canep.2014.09.006

[213] Xie Q , Bai Q , Zou LY , et al. Genistein inhibits DNA methylation and increases expression of tumor suppressor genes in human breast cancer cells. Genes Chromosomes Cancer. 2014;53:422‐431. 24532317 10.1002/gcc.22154

[214] Rice JC , Allis CD . Histone methylation versus histone acetylation: new insights into epigenetic regulation. Curr Opin Cell Biol. 2001;13:263‐273. 11343896 10.1016/s0955-0674(00)00208-8

[215] Ergün S , Ulasli M , Igci YZ , et al. The association of the expression of miR‐122‐5p and its target ADAM10 with human breast cancer. Mol Biol Rep. 2015;42:497‐505. 25318895 10.1007/s11033-014-3793-2

[216] De La Parra C , Castillo‐Pichardo L , Cruz‐Collazo A , et al. Soy isoflavone genistein‐mediated downregulation of miR‐155 contributes to the anticancer effects of genistein. Nutr Cancer. 2016;68:154‐164. 26771440 10.1080/01635581.2016.1115104 PMC4936403

[217] Venkatalakshmi P , Vadivel V , Brindha P . Role of phytochemicals as immunomodulatory agents: a review. Int J Green Pharm. 2016;10:1‐18.

[218] Yadav VS , Mishra KP , Singh DP , Mehrotra S , Singh VK . Immunomodulatory effects of curcumin. Immunopharmacol Immunotoxicol. 2005;27:485‐497. 16237958 10.1080/08923970500242244

[219] Ginwala R , McTish E , Raman C , et al. Apigenin, a natural flavonoid, attenuates EAE severity through the modulation of dendritic cell and other immune cell functions. J Neuroimmune Pharmacol. 2016;11:36‐47. 26040501 10.1007/s11481-015-9617-x PMC4857760

[220] Zhang R , Li Y , Wang W . Enhancement of immune function in mice fed high doses of soy daidzein. Nutr Cancer. 1997;29:24‐28. 9383780 10.1080/01635589709514597

[221] Yi J , Chen C , Liu X , et al. Radioprotection of EGCG based on immunoregulatory effect and antioxidant activity against 60Coγ radiation‐induced injury in mice. Food Chem Toxicol. 2020;135:111051. 31837348 10.1016/j.fct.2019.111051

[222] Wang J , Zhang Q , Jin S , He D , Zhao S , Liu S . Genistein modulate immune responses in collagen‐induced rheumatoid arthritis model. Maturitas. 2008;59:405‐412. 18499367 10.1016/j.maturitas.2008.04.003

[223] Jia Z , Chen A , Wang C , et al. Amelioration effects of Kaempferol on immune response following chronic intermittent cold‐stress. Res Vet Sci. 2019;125:390‐396. 31412308 10.1016/j.rvsc.2019.08.012

[224] So FV , Guthrie N , Chambers AF , Moussa M , Carroll KK . Inhibition of human breast cancer cell proliferation and delay of mammary tumorigenesis by flavonoids and citrus juices. Nutr Cancer. 2009;5581:167‐181 10.1080/01635589609514473 8875554

[225] Soo YC , Sung MK , Kim NH , Jang JO , Go EJ , Lee HJ . Inhibition of P‐glycoprotein by natural products in human breast cancer cells. Arch Pharm Res. 2005;28:823‐828. doi:10.1007/BF02977349 16114498

[226] Molnár J , Engi H , Hohmann J , et al. Reversal of multidrug resistance by natural substances from plants. Curr Top Med Chem. 2010;10:1757‐168. 20645919 10.2174/156802610792928103

[227] Mitra S , Dash R . Natural products for the management and prevention of breast cancer. Evid Based Complement Alternat Med. 2018;2018:1‐23. 10.1155/2018/8324696 PMC5846366 29681985

[228] Xue X , Liang XJ . Overcoming drug efflux‐based multidrug resistance in cancer with nanotechnology. Chin J Cancer. 2012;31:100‐109. doi:10.5732/cjc.011.10326 22237039 PMC3777470

[229] Castro AF , Altenberg GA . Inhibition of drug transport by genistein in multidrug‐resistant cells expressing P‐glycoprotein. Biochem Pharmacol. 1997;53:89‐93. 8960067 10.1016/s0006-2952(96)00657-0

[230] Yarden Y . The EGFR family and its ligands in human cancer: Signalling mechanisms and therapeutic opportunities. Eur J Cancer. 2001;37:3‐8. 11597398 10.1016/s0959-8049(01)00230-1

[231] Starok M , Preira P , Vayssade M , Haupt K , Salomé L , Rossi C . EGFR inhibition by curcumin in cancer cells: a dual mode of action. Biomacromolecules. 2015;16:1634‐1642. 25893361 10.1021/acs.biomac.5b00229

[232] Chandrika BB , Steephan M , Kumar TRS , Sabu A , Haridas M . Hesperetin and Naringenin sensitize HER2 positive cancer cells to death by serving as HER2 tyrosine kinase inhibitors. Life Sci. 2016;160:47‐56. 27449398 10.1016/j.lfs.2016.07.007

[233] Maashi MS , Al‐Mualm M , al‐Awsi GRL , et al. Apigenin alleviates resistance to doxorubicin in breast cancer cells by acting on the JAK/STAT signaling pathway. Mol Biol Rep. 2022;49:8777‐8784. 35804214 10.1007/s11033-022-07727-0

[234] Kim S , Lee TJ , Leem J , Choi KS , Park JW , Kwon TK . Sanguinarine‐induced apoptosis: generation of ROS, down‐regulation of Bcl‐2, c‐FLIP, and synergy with TRAIL. J Cell Biochem. 2008;104:895‐907. 18189268 10.1002/jcb.21672

[235] Li Y , Zhao H , Wang Y , et al. Isoliquiritigenin induces growth inhibition and apoptosis through downregulating arachidonic acid metabolic network and the deactivation of PI3K/Akt in human breast cancer. Toxicol Appl Pharmacol. 2013;272:37‐48. 23747687 10.1016/j.taap.2013.05.031

[236] Bimonte S , Cascella M , Barbieri A , Arra C , Cuomo A . Current shreds of evidence on the anticancer role of EGCG in triple negative breast cancer: an update of the current state of knowledge. Infect Agent Cancer. 2020;5:1‐6. 10.1186/s13027-020-0270-5 PMC6954554 31938038

[237] El‐Hafeez AAA , Khalifa HO , Mahdy EAM , Sharma V , Hosoi T , Ghosh P . Anticancer effect of nor‐wogonin (5, 7, 8‐trihydroxyflavone) on human triple‐negative breast cancer cells via downregulation of TAK1, NF‐κB, and STAT3. Pharmacol Rep. 2019;71:289‐298. 30826569 10.1016/j.pharep.2019.01.001 PMC6536637

